# Recent Advances in Polymer-Coated Metal and Metal Oxide Nanoparticles: From Design to Promising Applications

**DOI:** 10.3390/nano15221744

**Published:** 2025-11-20

**Authors:** Refia Atik, Rafiqul Islam, Melissa Ariza Gonzalez, Pailinrut Chinwangso, T. Randall Lee

**Affiliations:** 1 Department of Chemistry, University of Houston, 4800 Calhoun Road, Houston, TX 77204-5003, USA; 2 Texas Center for Superconductivity, University of Houston, 4800 Calhoun Road, Houston, TX 77204-5003, USA

**Keywords:** polymer-coated nanoparticles, hybrid nanomaterials, metal and metal oxide nanoparticles, core-shell structures, surface functionalization, nanocomposites, biomedical applications, biosensing, environmental remediation, energy storage and conversion

## Abstract

The integration of polymer coatings with metal and metal oxide nanoparticles represents a significant advancement in nanotechnology, enhancing the stability, biocompatibility, and functional versatility of these materials. These enhanced properties make polymer-coated nanoparticles key components in a wide range of applications, including biomedicine, catalysis, environmental remediation, electronics, and energy storage. The unique combination of polymeric materials with metal and metal oxide cores results in hybrid structures with superior performance characteristics, making them highly desirable for various technological innovations. Polymer-coated metal and metal oxide nanoparticles can be synthesized through various methods, such as grafting to, grafting from, grafting through, in situ techniques, and layer-by-layer assembly, each offering distinct control over nanoparticle size, shape, and surface functionality. The distinctive contribution of this review lies in its systematic comparison of polymer-coating synthesis approaches for different metal and metal oxide nanoparticles, revealing how variations in polymer architecture and surface chemistry govern their stability, functionality, and application performance. The aim of this paper is to provide a comprehensive overview of the current state of research on polymer-coated nanoparticles, including metals such as gold, silver, copper, platinum, and palladium, as well as metal oxides like iron oxide, titanium dioxide, zinc oxide, and aluminum oxide. This review highlights their design strategies, synthesis methods, characterization approaches, and diverse emerging applications, including biomedicine (e.g., targeted drug delivery, gene delivery, bone tissue regeneration, imaging, antimicrobials, and therapeutic interventions), environmental remediation (e.g., antibacterials and sensors), catalysis, electronics, and energy conversion.

## 1. Introduction

The advent of nanotechnology has created significant opportunities for the development of complex materials with tailored features [1,2,3,4,5]. Among these, polymer-coated metal and metal oxide nanoparticles have garnered considerable attention due to their unique combination of characteristics. These hybrid nanostructures, which integrate the beneficial properties of polymers with those of inorganic nanoparticles, exhibit enhanced stability, biocompatibility, and functional versatility, making them particularly appealing for a broad range of applications [6,7,8].

The unique physical and chemical properties of metal and metal oxide nanoparticles, such as their optical behavior, magnetic characteristics, and catalytic activity, make them indispensable for numerous technological advancements [9,10,11]. However, their inherent instability and tendency to aggregate in various environments pose substantial challenges [12]. These limitations are effectively addressed by polymer coatings, which form a stabilizing shell that prevents agglomeration while introducing additional functionalities such as improved solubility and environmental responsiveness [13,14,15,16].

The importance of polymer coatings in enhancing the stability and functionality of metal and metal oxide nanoparticles cannot be overstated. Beyond mitigating oxidation and agglomeration, polymer shells improve biocompatibility for biomedical applications and provide a versatile platform for further functionalization [17,18,19,20,21]. Functionalization of polymer coatings to include specific functional groups enables responsiveness to environmental stimuli such as pH, temperature, or light, facilitating applications like selective catalysis or controlled drug release [22,23,24]. For instance, in antimicrobial applications, polymer coatings enable the controlled release of metal ions with potent antibacterial properties. This controlled release is vital for maintaining prolonged antimicrobial activity while minimizing toxicity to human cells, making polymer-coated nanoparticles ideal for use in wound dressings, coatings for medical devices, and other healthcare applications [25,26,27,28,29].

In biosensing, polymer coatings play a critical role in functionalizing nanoparticles with specific ligands or antibodies, enabling highly selective and sensitive detection of biomolecules. This is particularly valuable for precisely identifying low-abundance biomarkers in the early stages of diseases [30,31,32]. In environmental remediation, polymer-coated metal and metal oxide nanoparticles are used to degrade organic pollutants [33,34]. The polymer layer prevents nanoparticle agglomeration and enhances their dispersibility in aqueous environments, ensuring reliable and efficient contaminant removal. Additionally, polymer coatings can be tailored to respond to environmental stimuli, further improving nanoparticle performance under diverse conditions [35,36].

Polymer coatings also play an essential role in catalysis by stabilizing metal and metal oxide nanoparticles, preventing aggregation, and facilitating the selective binding of reactants. This enhances catalytic efficiency in reactions such as oxidation and hydrogenation [37,38]. Similarly, in sensors and electronics, polymer coatings protect nanoparticle cores, form conductive polymer layers, and enable the creation of flexible, robust, and highly sensitive sensors [39]. In energy conversion and storage device applications, such as fuel cells, polymer coatings enhance nanoparticle performance and stability [40,41].

**Figure 1:**
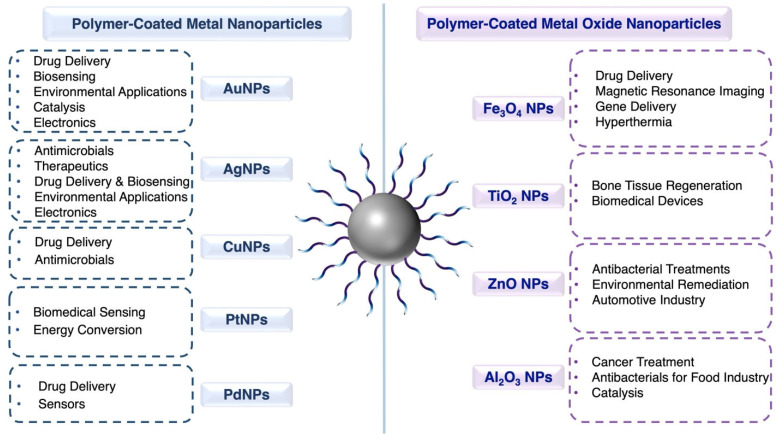
Overview of different types of polymer-coated metal and metal oxide nanoparticles and their potential applications as discussed in this review.

In addition to these application areas, polymer-coated metal and metal oxide nanoparticles hold potential for an even broader range of applications. This potential can be realized by designing appropriate polymer systems, selecting suitable metal or metal oxide nanoparticles, and employing precise coating methods tailored to specific applications, as illustrated in Figure 1.

This review aims to provide a comprehensive overview of the current state of research on hybrid nanomaterials (HNs), focusing on metal and metal oxide nanoparticles coated with custom-designed polymers. Emphasis is placed on recent advances in their synthesis, coating methodologies, characterization techniques, and applications. By highlighting both opportunities and challenges in this rapidly evolving field, we aim to guide future research toward the development of more efficient, scalable, and sustainable nanomaterials for a wide range of technological applications.

## 2. Modification of Metal and Metal Oxide Nanoparticles with Polymer Coatings

The synthesis of polymer-coated metal and metal oxide nanoparticles represents a significant advancement in nanotechnology, combining the versatile properties of polymers with the unique functionalities of metal and metal oxide nanoparticles [6,42,43]. This integration has enabled new developments in various fields, such as biomedicine, environmental science, and electronics, by enhancing stability, biocompatibility, and tunable surface characteristics [44,45,46].

The synthetic approaches for creating these nanocomposites can be broadly categorized into physical adsorption (physisorption) and chemical adsorption (chemisorption). Physisorption involves weak, non-covalent interactions that enable polymer molecules to adhere to the surface of metal or metal oxide nanoparticles. These interactions are typically governed by van der Waals forces, hydrogen bonding, or electrostatic attractions. Since physisorption is a reversible process, the adsorbed polymer can be easily detached from the nanoparticle surface by adjusting solvent polarity, temperature, or pressure. As a result, polymer coatings formed through physisorption tend to be less stable and more susceptible to external influences, making them particularly useful in applications requiring reversible or dynamic coatings. This method is often preferred when mild conditions are required to preserve the structural integrity and functional properties of both the polymer and the nanoparticle [47,48,49].

In contrast, chemisorption involves the formation of strong, often covalent, bonds between the polymer molecules and the nanoparticle surface. This process typically occurs when the polymer contains functional groups capable of chemically interacting with metal or metal oxide surfaces. Unlike physisorption, chemisorption is generally irreversible, producing a more stable and durable polymer coating [47,50]. The strong binding interactions in chemisorption ensure that the polymer remains attached to the nanoparticle even under harsh conditions, such as high temperatures or exposure to solvents. Several strategies can be employed for chemisorption, including the “grafting from” method (surface-initiated polymerization), the “grafting to” method (post-synthesis polymer coating), the “grafting through” method, and the in situ approach. Each of these methods follows distinct methodologies and has specific applications, which will be discussed in the following sections. Additionally, layer-by-layer (LbL) assembly is another widely used technique for fabricating hybrid nanomaterials. However, depending on the nature of the interactions involved, LbL assembly may involve either physisorption or chemisorption, as will be explained in its respective section in this review.

To successfully implement these coating strategies, it is also essential to consider specific polymerization techniques, such as atom transfer radical polymerization (ATRP), reversible addition-fragmentation chain transfer (RAFT) polymerization, ring-opening polymerization (ROP), anionic polymerization, cationic polymerization, free radical (chain-growth) polymerization, and oxidative chemical polymerization. Furthermore, the choice of polymerization technique and its initiation method must be carefully optimized to achieve efficient and stable nanocomposites tailored for specific applications.

### 2.1. Grafting to Method (Post-Synthesis Polymer Coating)

**Figure 2:**
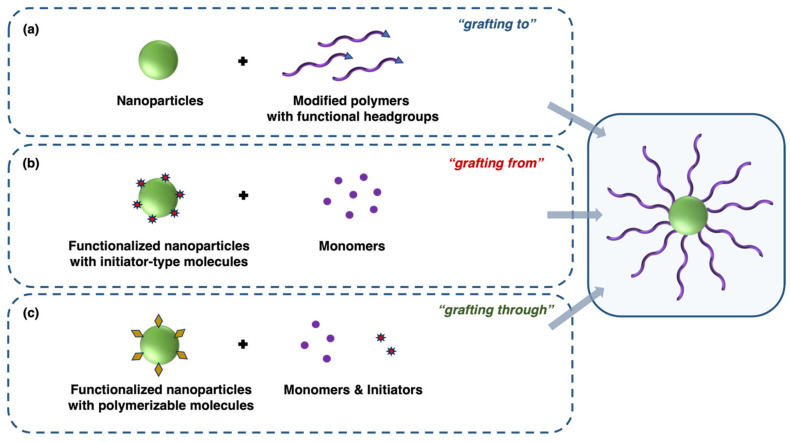
Schematic illustration of grafting methods on nanoparticles: (**a**) grafting to, (**b**) grafting from, and (**c**) grafting through.

The “grafting to” method is a widely used approach for creating polymer-grafted surfaces, involving the chemical attachment of preformed polymer chains to a functionalized surface. In this method, which is shown in Figure 2a, a grafted layer is formed by anchoring end-functionalized polymers to reactive sites on a substrate. The process begins with the synthesis of polymer chains possessing specific functional groups at the end of the chain, typically via standard polymerization methods such as anionic polymerization, ring-opening polymerization, or living/controlled radical polymerization. Once synthesized, these polymers are introduced to a surface containing complementary reactive groups. The polymer’s functional groups react chemically with the surface’s reactive sites to form a stable attachment [51,52].

One of the primary advantages of the “grafting to” method is the ability to precisely control the properties of the grafted layer by pre-determining the molecular weight, composition, and architecture of the polymer chains before grafting. This enables fine-tuning of surface characteristics such as wettability, biocompatibility, and chemical resistance. However, a significant limitation of this approach is steric hindrance, which can restrict grafting density. As more polymer chains attach to the surface, steric effects may hinder further attachment, thereby limiting the thickness of the polymer and potentially affecting the functional performance of the grafted surface in various applications [53].

Despite these limitations, the “grafting to” method remains widely employed in fields such as biomaterial surface modification, anti-fouling coatings, and the development of stimuli-responsive interfaces. Its ability to allow for the pre-synthesis and characterization of polymers prior to attachment makes it particularly advantageous in applications where precise control over polymer properties is critical [54,55].

### 2.2. Grafting from Method (Surface-Initiated Polymerization)

The “grafting from” method, also known as surface-initiated polymerization, is a widely recognized technique used for the synthesis of polymer-grafted surfaces, where polymer chains are directly grown from initiator sites on a substrate. Unlike the “grafting to” method, in which preformed polymer chains are attached to a surface, the “grafting from” method facilitates polymerization directly at the surface, enabling higher grafting densities and the formation of thicker polymer layers, as can be seen in Figure 2b [56].

In this approach, initiator molecules capable of initiating polymerization are first immobilized on the substrate surface to functionalize it. These initiators can be designed for various polymerization techniques, including anionic, cationic, ring-opening polymerization, and controlled/living radical polymerization [57,58,59]. Once surface functionalization is complete, monomers are introduced into the system, and the surface-bound initiators directly initiate polymerization. As a result, polymer chains grow outward from the surface, forming a dense and uniform polymer brush [51].

One of the primary advantages of the “grafting from” method is its ability to achieve high grafting densities, which distinguishes it from the “grafting to” method. This results in the formation of more homogeneous and thicker polymer layers, making it particularly beneficial for applications requiring strong surface coatings with enhanced mechanical and chemical properties [56]. Additionally, the “grafting from” method allows for the use of a wide range of polymerization techniques, enabling the synthesis of complex polymer structures such as gradient polymers, block copolymers, and hyperbranched polymers. By modifying reaction parameters such as monomer concentration, reaction time, and temperature, surface-initiated polymerization provides precise control over polymer brush thickness and architecture [60,61,62,63].

However, the “grafting from” method also presents certain challenges. In order to prevent undesirable side reactions such as termination or chain transfer, which can lead to non-uniform grafting, the polymerization process must be carefully controlled. Furthermore, since the grafted polymers remain attached to the surface, their characterization can be more complex compared to the “grafting to” method, where free polymer chains can be analyzed more easily [64,65].

Despite these challenges, the “grafting from” method remains a highly effective and versatile approach for fabricating polymer-grafted surfaces with high grafting densities and tailored properties. Its advantages in achieving thicker, more homogeneous layers and the ability to construct intricate polymer architectures make it a valuable strategy in advanced material design, provided that the polymerization process is carefully monitored and controlled.

### 2.3. Grafting Through Method

The polymer synthesis technique known as the “grafting through” method forms polymer-grafted structures by polymerizing pre-functionalized monomers through a backbone or template. In contrast to the “grafting from” and “grafting to” techniques, the “grafting through” method involves the polymerization of macromonomers, where the polymerizable group is integrated into the monomer, thereby enabling the formation of densely grafted polymers or polymer networks, as illustrated in Figure 2c. In this method, a macromonomer, which is a preformed polymer chain with a reactive polymerizable end-group, is introduced into a polymerization system [51].

The “grafting through” technique offers several advantages, particularly for synthesizing complex macromolecular structures and densely grafted polymers. By utilizing preformed macromonomers, the length, composition, and functionality of the grafts can all be precisely controlled prior to polymerization [66]. However, this method also has certain limitations. The synthesis of macromonomers is often labor-intensive and requires multiple steps to achieve the desired functionality. Additionally, due to their size and steric hindrance, macromonomers can present challenges during polymerization, potentially leading to uneven grafting or incomplete polymerization. Moreover, the incorporation of large macromonomers can slow down the polymerization process, making the “grafting through” method less efficient for producing very high molecular weight polymers or for applications where extremely high grafting densities are needed [67].

In summary, the “grafting through” method is a powerful approach for synthesizing densely grafted polymers with well-defined architectures and tailored properties. While it requires careful macromonomer synthesis and controlled polymerization conditions, it offers significant advantages for the development of advanced materials with specialized applications.

### 2.4. In Situ Approach

**Figure 3:**
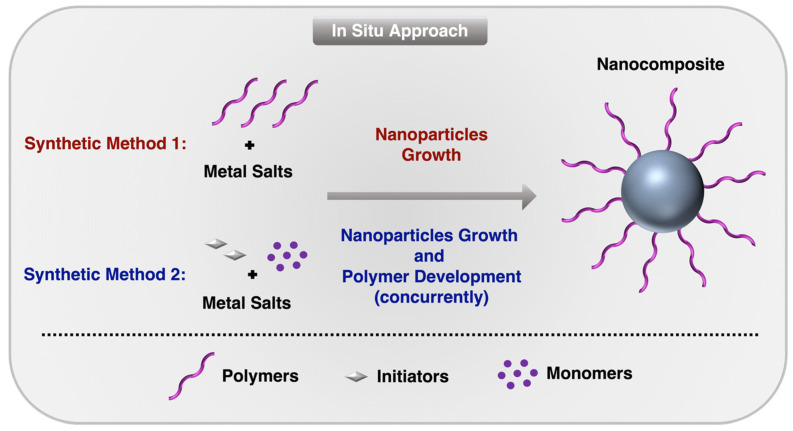
Simple schematic illustration of the in situ synthesis method for hybrid polymer-nanoparticle nanocomposites.

The in situ approach involves the simultaneous synthesis of metal nanoparticles from metal precursors and their encapsulation or coating within a polymer matrix. This process enables the formation of well-defined, stable, and functionalized nanoparticles with enhanced properties by integrating metal ion reduction with polymerization or polymer assembly in a single step [68]. This method is particularly useful for producing hybrid materials with tailored functionalities, where the metal core and polymer shell interact synergistically. As illustrated in Figure 3, in the in situ approach, a polymer or polymerizable monomer is dissolved in a reaction medium along with metal precursors, such as metal salts (e.g., silver nitrate, gold chloride, or palladium acetate). The metal ions are then reduced to form nanoparticles, typically through chemical reduction using a reducing agent (e.g., sodium borohydride) or via alternative methods such as thermal, photochemical, or electrochemical reduction. Concurrently, the polymer encapsulates the forming nanoparticles either by self-assembly around them or through polymerization in the presence of the metal [69,70].

The in situ approach offers several advantages, foremost among them being the one-step production of well-dispersed nanoparticles with controlled size, morphology, and surface functionality. Due to their in situ formation, the polymer coating and metal core are closely integrated, which enhances nanoparticle stability and functionality. Furthermore, the incorporation of various functional groups into the polymer shell enables additional modifications or targeting capabilities, which is particularly beneficial for catalytic and biomedical applications. However, the in situ approach requires precise control over multiple parameters to achieve the desired nanoparticle size, uniformity, and coating quality. The simultaneous reduction in metal ions and polymerization or self-assembly of the polymer must be carefully balanced, making the process challenging to optimize. The final properties of the nanoparticles are determined by a number of factors, including the concentration of the metal precursor, the type of reducing agent, the nature of the polymer or monomer, and key reaction conditions such as temperature, pH, and solvent composition [71,72].

In summary, the in situ approach provides a robust and versatile strategy for synthesizing polymer-coated metal nanoparticles, enabling the production of stable, functionalized hybrid materials in a single step. Despite the challenges associated with process control, this approach remains highly attractive for a wide range of advanced applications.

### 2.5. Layer-by-Layer (LbL) Assembly

The layer-by-layer (LbL) self-assembly method is a widely used technique for fabricating multilayered films by alternately depositing oppositely charged materials, such as polyelectrolytes or nanoparticles, onto a substrate [45,73]. Depending on the nature of the interactions involved, the LbL process can proceed via physisorption or chemisorption. The process can be predominantly governed by physisorption if the layers are primarily held together by hydrogen bonds, electrostatic forces, or van der Waals interactions. In contrast, chemisorption occurs when covalent bonding takes place between layers or with the surface itself.

The LbL technique provides remarkable control over film characteristics, allowing the fabrication of nanoscale structures with tailored properties such as optical transparency, conductivity, or biocompatibility [74]. However, despite its advantages, the LbL process can be time-consuming, especially when multiple layers are required. Additionally, environmental factors such as pH and ionic strength can also have an impact on the stability of the films, potentially altering their properties over time.

However, LbL self-assembly remains a powerful tool in materials science and nanotechnology because it can produce highly ordered and multifunctional films with nanoscale precision. This method provides a versatile and precise approach for fabricating multilayer films with controlled properties, making it essential for advancing next-generation materials across various industries [75,76].

### 2.6. Specific Polymerization Methods

The methods for developing polymer shells around nanoparticles vary widely and often involve different energy sources, such as heat, light, or radiation, each of which can initiate or catalyze polymerization processes [77].

Thermal initiation involves the use of heat to generate free radicals from initiator molecules, which then initiate polymerization. Heat is the most commonly used energy source for the initiation of polymerization reactions. It can facilitate the thermal decomposition of initiators grafted onto nanoparticles, leading to polymer growth directly from the particle surface. For instance, polymer coatings around nanoparticles can be synthesized through thermally initiated polymerization of styrene or methyl methacrylate [78,79]. Another common initiation method is photo-initiation, which typically employs UV light to initiate polymerization via photo-initiators that generate radicals upon exposure to specific wavelengths. This method offers advantages in terms of temporal and spatial control of polymerization, allowing the formation of polymer coatings under mild conditions with tunable properties by adjusting the light exposure. Photo-initiation is widely used in the synthesis of hydrogel nanoparticles, where light-sensitive monomers are polymerized around a nanoparticle core [80,81]. Furthermore, high-energy radiation, such as gamma rays or electron beams, can initiate polymerization without chemical initiators. Radiation induces the generation of radicals directly from the monomers or nanoparticle surfaces, leading to polymer growth. This method is particularly useful in biomedical applications, as it enables simultaneous sterilization and polymerization [82,83,84,85]. These initiation methods provide flexibility and control over the polymerization process, facilitating the synthesis of polymer-coated nanoparticles with desired properties.

Among the specific polymerization methods, ATRP, RAFT polymerization, ROP, anionic polymerization, cationic polymerization, free radical (chain-growth) polymerization, and oxidative chemical polymerization are particularly noteworthy.

ATRP is a controlled radical polymerization technique that enables precise control of molecular weight and polymer architecture using a transition metal catalyst to mediate the polymerization process. This method allows for the growth of polymer chains from functionalized nanoparticle surfaces, producing well-defined polymers with narrow molecular weight distributions. It is also capable of synthesizing block copolymers, making it highly versatile for a wide range of monomers and specific functionalities. The ability to pause and resume the polymerization process adds further flexibility. However, the use of transition metal catalysts, which can be costly and challenging to remove from the final product, is a notable drawback [86,87].

RAFT polymerization, another controlled radical polymerization method, uses RAFT agents to mediate polymerization, offering excellent control over polymer architecture and molecular weight. This technique is advantageous for producing complex polymer structures, such as block copolymers and star polymers, and is compatible with a wide range of monomers. It is particularly useful for creating stimuli-responsive polymers, such as thermoresponsive or pH-responsive coatings on nanoparticles. RAFT polymerization can be conducted under relatively mild conditions, making it accessible and practical. However, the necessity for specific chain transfer agents, which can be expensive or difficult to synthesize, and the challenge of removing residual agents from the final product, are notable disadvantages. RAFT is extensively used for producing block copolymers, nanomaterials, and polymers for biomedical applications, such as drug delivery and tissue engineering [88,89,90].

ROP is a chain-growth polymerization technique in which cyclic monomers undergo ring-opening reactions to form polymer chains. It is particularly effective for producing biodegradable and biocompatible polymers, such as polyesters, polyamides, and polypeptides. This method accommodates a broad range of cyclic monomers and allows for precise control over molecular weight and polymer architecture. However, ROP requires specific catalysts that can be sensitive to moisture and impurities, adding complexity to the process. ROP is commonly employed in the synthesis of biodegradable polymer coatings on nanoparticles for drug delivery applications [91,92].

Anionic polymerization involves the use of an anionic initiator to start the polymerization process, offering exceptional control over molecular weight and polymer architecture. This method is known for its ability to produce polymers with very narrow molecular weight distributions and for facilitating living polymerization, which enables the formation of block copolymers and other complex architectures. However, the process is highly sensitive to moisture and impurities, and only certain monomers are suitable for this type of polymerization [93,94].

Cationic polymerization employs a cationic initiator to start the polymerization process, making it suitable for monomers with electron-donating groups. This method is characterized by rapid polymerization rates and the ability to control polymer stereochemistry, which is beneficial for producing polymers with specific properties. However, similar to anionic polymerization, cationic initiators can be highly sensitive and reactive, and the method is limited to specific types of monomers [95,96].

Free radical polymerization, a widely used addition (chain-growth) polymerization technique, involves the use of free radicals to initiate and propagate polymerization. This method is highly versatile, allowing for the polymerization of a broad range of monomers. It is also simple and cost-effective, requiring relatively simple and inexpensive equipment. Free radical polymerization often proceeds rapidly, making it suitable for large-scale production. However, compared to more advanced methods, it offers less control over molecular weight distribution and polymer architecture. Termination reactions can also occur, leading to premature chain termination [97,98].

Lastly, oxidative chemical polymerization is widely used to synthesize conducting polymers such as polyaniline, polypyrrole, and polythiophene. In this method, monomers undergo polymerization in the presence of an oxidizing agent, which triggers a redox reaction leading to polymer chain formation. The oxidizing agent initiates the reaction by converting monomer molecules into radical cations, which subsequently react with additional monomer units to propagate the polymer chain. Typically, oxidizing agents such as ferric chloride or ammonium persulfate are used to oxidize the monomer, generating radical cations that link with other monomer units to continue the polymerization process. Polymers produced via oxidative chemical polymerization find extensive applications in sensors, energy storage, electronics, and corrosion prevention [99].

In summary, the synthesis of polymer-coated metal and metal oxide nanoparticles encompasses a variety of techniques, each offering distinct advantages that can be leveraged to achieve specific material properties. By carefully selecting the appropriate polymerization method and polymer type, researchers can design advanced nanocomposites tailored for a wide range of innovative applications, thereby advancing nanotechnology and its diverse applications.

## 3. Structures, Properties, and Applications of Polymer-Coated Metal Nanoparticles

Metal nanoparticles coated with polymers constitute a significant class of hybrid nanomaterials that integrate the tunable properties of polymeric shells with the distinctive physicochemical characteristics of metal cores. These nanostructures exhibit unique features that make them valuable for diverse applications, including environmental remediation, biomedical devices, catalysis, and sensing technologies [6,39,100].

A key property of metal nanoparticles is their ability to exhibit localized surface plasmon resonance (LSPR), wherein the collective oscillation of conduction electrons resonates with incident light, leading to strong absorption and scattering at specific wavelengths. Notably, polymer coatings can influence LSPR by altering the local dielectric environment, thereby enabling precise modulation of the nanoparticles’ optical properties for targeted applications [101].

The following sections will explore the structures, properties, and applications of various polymer-coated metal nanoparticles, including those based on gold, silver, copper, platinum, and palladium (Table 1). In particular, the discussion will focus on how polymer-metal interactions enhance nanoparticle performance in various applications such as drug delivery, biosensing, photothermal therapy, antimicrobial treatments, environmental remediation, catalysis, and electronics.

### 3.1. Gold Nanoparticles (AuNPs)

Gold nanoparticles (AuNPs) have garnered significant attention in nanotechnology due to their exceptional physicochemical properties and diverse applications. These nanoparticles are particularly valued for their unique optical characteristics, notably their strong absorption and light scattering arising from LSPR. This property enhances their utility in biomedical and technological applications. Furthermore, AuNPs exhibit excellent biocompatibility and chemical stability, making them suitable for complex biological environments [124,125,126].

The properties and applications of AuNPs can be considerably affected by their size and shape. AuNPs can adopt various shapes, including spheres, rods, stars, and cubes, each displaying distinct electronic and optical properties [124]. For instance, spherical AuNPs are widely utilized in diagnostic applications owing to their tunable and strong LSPR, which enhances biosensor sensitivity [127]. Due to their anisotropic shape, gold nanorods exhibit two plasmon resonance peaks—longitudinal and transverse—making them particularly useful for photothermal therapy and imaging applications [128].

Beyond simple geometries, AuNPs can be engineered into more complex nanoshell structures, where a dielectric core (e.g., silica) is coated with a thin layer of gold shell. These core–shell architectures exhibit tunable plasmonic properties, with their resonances adjustable by modifying the core size and shell thickness. Nanoshells are particularly beneficial in photothermal therapy as they efficiently absorb near-infrared (NIR) light and convert it into heat, enabling the selective destruction of cancer cells [129,130].

The incorporation of polymer coating onto AuNPs further enhances their stability and functionality. Polymer-coated AuNPs have found applications in drug delivery, biosensing, environmental remediation, catalysis, and electronics. The following sections discuss recent advances in these applications.

#### 3.1.1. Polymer-Coated AuNPs in Targeted Drug Delivery

The polymer shell serves as a versatile platform for the attachment of targeting ligands, drugs, and other functional groups, which is crucial for applications requiring precise control over nanoparticle interactions with biological systems [126]. In targeted drug delivery, functionalized polymer coatings can be designed to recognize and bind to specific cellular receptors, ensuring that therapeutic agents are selectively delivered to target cancer cells while minimizing off-target effects.

**Figure 4:**
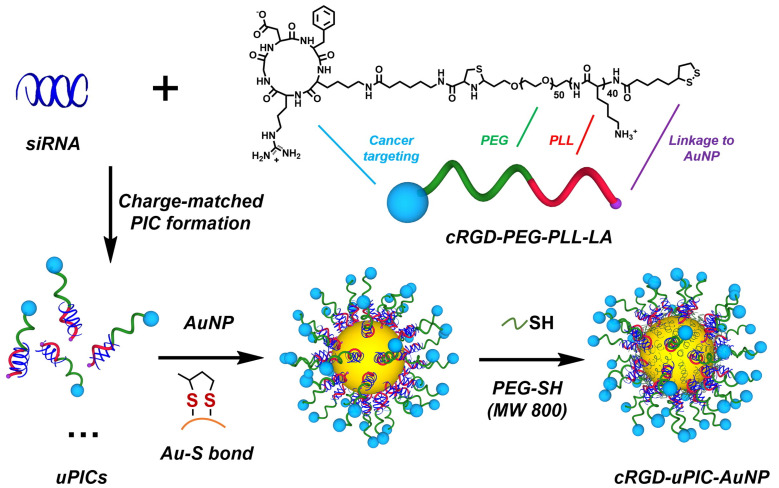
Schematic illustration of the preparation of cRGD-uPIC-AuNP. Initially, a single pair of siRNA and cRGD-PEG-PLL, modified with lipoic acid (LA) at the ω-end (cRGD-PEG-PLL-LA), was used to generate monodispersed cRGD-functionalized uPICs. The cRGD moiety facilitated cancer cell targeting, while the LA moiety enabled conjugation to the AuNP surface. In the second step, the uPICs were conjugated onto the AuNP core via double Au–S bonds. A short PEG chain was then grafted onto the nanoparticles to enhance dispersion and stability, yielding monodispersed cRGD-uPIC-AuNPs. Reproduced with permission from Ref. [102]. Copyright 2016 Elsevier.

Yi et al. developed cyclic Arg-Gly-Asp (RGD) peptide-functionalized unimer polyion complex (uPIC)-assembled AuNPs (cRGD-uPIC-AuNPs) for the targeted systemic delivery of small interfering RNA (siRNA) to cervical cancer models [102]. The synthesis involved a two-step process: (i) formation of uPICs by complexing siRNA with cRGD-PEG-PLL-LA block copolymers, which is cyclic Arg-Gly-Asp peptide-attached poly(ethylene glycol)-*block*-poly(L-lysine) customized with lipoic acid (LA) at the ω-end, via anionic polymerization, followed by (ii) functionalization of AuNPs through a “grafting to” approach, as illustrated in Figure 4. LA moiety at the polymer terminus played a critical role in forming a stable Au-S bond, enabling the conjugation of polymer-siRNA complexes onto the AuNP surface. The thiol head group facilitated this conjugation, enhancing the stability and performance of the nanocarrier. Additionally, short PEG chains (PEG-SH 800) were incorporated to prolong circulation time and improve biocompatibility in vivo.

These cRGD-uPIC-AuNPs were specifically designed to target α_v_β_3_/α_v_β_5_ integrins, which are overexpressed on endothelial and tumor cells, thereby enhancing tumor accumulation and cellular uptake. Several characterization techniques were employed to confirm the successful synthesis and functionalization of these nanoparticles. Analytical ultracentrifugation (AUC) was used to verify the molecular weight of the uPICs, while fluorescence correlation spectroscopy (FCS) confirmed their formation. Zeta potential measurements indicated successful surface modification, whereas dynamic light scattering (DLS) and transmission electron microscopy (TEM) revealed that the uPIC-AuNPs had a uniform size of approximately 40 nm.

In vitro studies demonstrated that cRGD-uPIC-AuNPs exhibited significantly higher uptake in HeLa cells due to their specific binding to integrin receptors. Compared with non-targeted controls, this enhanced uptake led to improved gene silencing of the E6 oncogene. In vivo experiments further confirmed that these targeted nanoparticles accumulated preferentially in tumors, leading to significant tumor growth inhibition without causing systemic toxicity. These findings highlight the potential of size-engineered nanocarriers for efficient, targeted siRNA delivery and cancer therapy.

While some research has focused on using peptide-functionalized AuNPs for targeted delivery systems, Brazzale et al. explored an alternative approach by developing pH-responsive AuNPs to enhance the selective targeting of cancer cells for therapeutic delivery [103]. The coating of these AuNPs consists of a dual-polymer system: a pH-responsive polymer that exposes the targeting ligand in the acidic environment typical for tumors, and a folate-functionalized PEG polymer that facilitates active targeting. The purpose of this “hide-and-reveal” mechanism is to improve the selectivity and efficacy of drug delivery systems in cancer treatment. By possibly using the “grafting to” method [51,52], these nanoparticles were functionalized with folate-PEG_2kDa_-SH (FA-PEG_2kDa_-SH), a PEG chain containing a folate-targeting ligand, and lipoyl-poly[2-(methacryloyloxy)ethyl-3-chloro-4-hydroxybenzoate]-*b*-[glycerol methacrylate] (lipoyl-[(MCH)_26_-*b*-(GMA)_53_]), a diblock copolymer synthesized by RAFT polymerization. This polymer undergoes conformational shrinkage at acidic pH (pH 6.5), which is a characteristic of tumor environments, thereby exposing the folate ligands and enabling receptor-mediated endocytosis. Additionally, a fluorescently labeled polymer, bodipy FL-PEG_2kDa_-SH (Bdp-PEG_2kDa_-SH), was synthesized by conjugating NH_2_-PEG_2kDa_-SH to an N-hydroxysuccinimidyl ester-activated bodipy FL dye. This polymer allowed for tracking the nanoparticles during biological investigations, facilitating the visualization and quantification of their cellular uptake. The pH-responsive behavior of this hybrid system was confirmed through various characterization techniques. TEM analysis verified size changes and provided direct visualization of the polymeric coating on the AuNPs. Zeta potential measurements further evaluated the surface charge of the nanoparticles, revealing a slight decrease in zeta potential at acidic pH, consistent with the predicted conformational changes in the pH-responsive polymer. To track the pH-responsive behavior, fluorescence quenching experiments were conducted. At lower pH levels, the polymer collapsed, bringing the fluorescent dye closer to the gold surface and reducing fluorescence intensity. Furthermore, cellular uptake studies revealed significantly higher uptake in cancer cells under acidic conditions compared to normal physiological environment (pH 7.4), confirming the effectiveness of “hide-and-reveal” strategy. The nanoparticles exhibited good stability and low toxicity in KB and MCF-7 cell lines. This study provides a proof-of-concept for utilizing pH-responsive AuNPs to selectively enhance drug delivery to specific cancer cells, thereby improving the efficacy and minimizing potential side effects of anticancer treatments.

**Figure 5:**
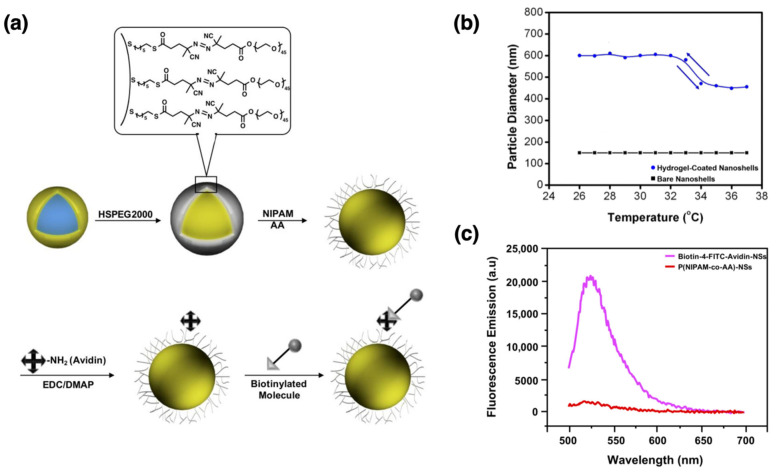
(**a**) Schematic representation of hydrogel growth on gold nanoshells via radical polymerization, followed by modification of the hydrogel periphery with avidin and biotin-4-FITC molecules. (**b**) Temperature-dependent hydrodynamic diameter of uncoated nanoshells (black squares) and hydrogel-coated nanoshells (blue circles). Arrows denote the heating and cooling scan directions, indicating the reversible size change of the hydrogel layer. (**c**) Fluorescence spectra of avidin-functionalized nanoparticles complexed with biotin-4-FITC (magenta) and the control sample (hydrogel-coated nanoshells, red). Reproduced with permission from Ref. [104]. Copyright 2018 by the authors. Published by MDPI under the Creative Commons CC BY license.

As a last example of drug delivery studies, Park et al. reported the development and characterization of heat-responsive hydrogel-coated gold-silica nanoshells for potential photothermally induced drug-delivery systems [104]. Gold nanoshells were selected as the core material due to their optical properties, particularly their tunable surface plasmon resonance (SPR), which can be adjusted to the NIR range. This tunability makes them highly suitable for biomedical applications, such as photothermal therapy. These nanoshells efficiently induce the collapse of the surrounding hydrogel layer and facilitate drug release by absorbing NIR light and converting it into heat. The nanoshells consist of a silica core (~120 nm) coated with a gold layer (~20 nm), imparting unique optical properties, including tunable SPR. As shown in Figure 5a, the nanoshells were functionalized with a surface-bound, custom-designed free radical initiator to generate a poly(*N*-isopropylacrylamide-*co*-acrylic acid) (P(NIPAM-*co*-AA)) hydrogel layer around the nanoshells using surface-initiated polymerization. Surface functionalization with avidin enabled further modification with fluorescent-tagged biotin (biotin-4-fluorescein or biotin-4-FITC) for imaging and monitoring. Scanning electron microscopy (SEM) confirmed successful encapsulation, while DLS revealed a decrease in nanoshell diameter with increasing temperature, indicating hydrogel layer collapse. UV–Visible spectroscopy showed an SPR peak at ~800 nm, which is well-suited for biomedical applications. The synthesis process involved encapsulating HSPEG2000 initiators around the nanoshells, followed by hydrogel layer formation using a “grafting from” method and free radical polymerization. The temperature-responsive behavior of the hydrogel-coated nanoshells was confirmed by DLS experiments, as shown in Figure 5b. Above the lower critical solution temperature (LCST), the hydrogel layer collapsed as the temperature increased from 25 °C to 40 °C, leading to a reduction in the hydrodynamic diameter of the nanoparticles. This reversible size change demonstrated the successful synthesis of temperature-responsive nanoshells capable of dynamically adjusting their size in response to temperature fluctuations. Figure 5c presents the fluorescence spectra of the hydrogel-coated nanoshells following their avidin modification and conjugation with biotin-4-fluorescein (biotin-4-FITC). The strong fluorescence emission observed in the avidin-biotin conjugated nanoshells, compared to the control group (nanoshells lacking avidin), confirmed successful bioconjugation. This functionalization enables the hydrogel-coated nanoshells to bind specifically to biotinylated molecules, making them suitable for targeted drug delivery applications.

Comparison of these polymer-coated AuNP systems reveals clear trade-offs between grafting approach, coating density, and responsive control. “Grafting to” strategies provide precise placement of targeting ligands and predictable surface chemistry, yet their lower grafting densities can limit long-term stability and reduce the strength of responsive transitions. In contrast, “grafting from” coatings form uniform, covalently anchored layers that resist desorption and enable reproducible heat-triggered drug release, although their compactness may slow diffusion of larger therapeutics. Systems maintaining intermediate shell densities and controlled cross-linking typically achieve the most favorable balance between mechanical robustness, permeability, and responsiveness. In practice, coatings that remain colloidally stable in physiological salt while permitting reversible swelling or collapse under external stimuli yield the highest therapeutic efficiency and reproducibility across studies [102,103,104].

#### 3.1.2. Polymer-Coated AuNPs in Biosensing

The functional groups of polymers on AuNPs can enhance the sensitivity and specificity of detection by facilitating the binding of specific biomolecules, enabling the detection of low-abundance analytes in biosensing applications. Takara et al. investigated glycopolymer-modified AuNPs as sensing agents for detecting concanavalin A (ConA), a mannose-binding protein [105]. They synthesized glycopolymers of poly(acrylamidophenyl α-mannose-*co*-acrylamide) (poly(AcMan-*r*-AAm)) via RAFT polymerization, achieving polymers with narrow polydispersities. These glycopolymers were subsequently functionalized with thiol groups to enable their binding to AuNPs using a process that can be considered a “grafting to” method [51,52]. By varying the mannose density through copolymerization and mixing with polyacrylamide, they developed glycopolymer-substituted AuNPs with controlled colloidal stability and molecular recognition properties.

In this design, mannose units on the glycopolymers specifically bound to ConA, facilitating its detection through multivalent binding effects. Nanoparticles with higher mannose densities exhibited stronger protein recognition and enhanced sensitivity. Several key characterization techniques were used to evaluate the glycopolymer-modified AuNPs. The synthesis and sugar content of the glycopolymers were confirmed via NMR spectroscopy. Molecular weight distributions with narrow polydispersity were assessed using size exclusion chromatography (SEC). According to DLS measurements, glycopolymer coating increased nanoparticle size to 55–70 nm. UV–Vis spectroscopy analysis indicated that colloidal stability under high-salinity conditions was greater when sugar density was lower. TEM verified the size and homogeneity of the particles. In immunochromatographic assays, these nanoparticles demonstrated notable biosensing capabilities, with higher sugar content enhancing sensitivity for ConA detection. These findings suggest that glycopolymer-modified AuNPs, synthesized via RAFT polymerization, are effective for ConA detection and are promising candidates for biosensing applications due to their molecular recognition properties and tunable stability.

Wang et al. reported colorimetric biosensors based on AuNPs hybridized with polymer coatings and functionalized with analyte recognition units [106]. They specifically utilized star-shaped PEG polymers with different arm numbers (four-arm and eight-arm PEG) to coat AuNPs using the “grafting to” method. These PEG-coated AuNPs were then functionalized with maleimide-functionalized sulfated heparin, which served as the recognition unit by binding to target proteins via electrostatic interactions. Characterization techniques confirmed the structural and functional properties of these nanoparticles. TEM analysis determined that the average diameter of the synthesized AuNPs was approximately 19 nm. After coating with PEG and further modification with heparin, hydrodynamic size and surface charge were analyzed using DLS and zeta potential measurements. The hydrodynamic diameter increased to approximately 74 nm after modification, while surface charge shifted from a more negative value following PEG coating to a less negative value after heparin attachment. Furthermore, Vis-NIR extinction spectroscopy was employed to monitor optical property changes, confirming the colloidal stability of the particles and the polymer coating’s role in preventing agglomeration. The study demonstrated that biosensor performance was highly dependent on the number of arms in the star-shaped PEG linker. For detecting specific proteins such as IL-8, nanoparticles coated with four-arm PEG linkers exhibited greater sensitivity than those coated with eight-arm PEG linkers. This difference was attributed to higher heparin load on four-arm PEG-coated nanoparticles, which facilitated stronger protein binding and more pronounced agglomeration, leading to a more notable optical response. However, linker topology had a less significant effect on sensor performance for proteins with a higher affinity for heparin, such as PDGF-BB, likely due to the inherently strong interaction between the protein and the heparin-coated nanoparticles. These findings highlight that the sensitivity of biosensors is significantly influenced by polymer coating topology, emphasizing the importance of interface design in the development of effective biosensing materials.

Overall, both glycopolymer- and PEG-based coatings illustrate how polymer topology and ligand density dictate biosensor performance by influencing surface recognition and colloidal stability. Glycopolymer shells provide strong, multivalent binding to lectins like ConA but can lose stability at high ionic strength when ligand density is excessive. PEG-based architectures offer greater stability and tunable surface charge, though sensitivity depends on polymer branching and accessibility of recognition sites. Four-arm PEG linkers yield stronger signals through higher analyte loading, whereas bulkier eight-arm structures limit diffusion and plasmonic coupling. In general, thin, flexible polymer layers with moderate ligand densities achieve the best balance between target affinity, signal response, and stability, emphasizing the importance of precise interface design in polymer-coated AuNP biosensors [105,106].

#### 3.1.3. Polymer-Coated AuNPs in Environmental Applications and Catalysis

Nanoparticles can be functionalized with selected polymers to catalyze the degradation of toxic substances or to capture and remove pollutants in environmental applications. Subair et al. developed polydopamine-modified membranes incorporating AuNPs for catalytic and environmental applications [107]. They employed a self-polymerization method of dopamine (DOPA), in which monomers undergo oxidation and spontaneously polymerize in an alkaline environment, forming a continuous polymeric coating without the need for initiators or catalysts. Basically, they grafted polyethyleneimine (PEI) for in situ gold nanoparticle adsorption on both DOPA and DOPA-PEI surfaces after modifying the polyethylene terephthalate (PET) track-etched membranes by the self-polymerization of DOPA. The membranes were characterized for their catalytic activity in reducing p-nitrophenol (PNP) and degrading organic dyes, specifically Rhodamine B and methylene blue. Several analytical techniques were employed to further examine the membranes and synthesized AuNPs. X-ray photoelectron spectroscopy (XPS) confirmed the chemical composition of the modified membranes, revealing the presence of AuNPs and the successful grafting of DOPA and PEI layers. Thermogravimetric analysis (TGA) assessed the thermal stability of the modified membranes, demonstrating the effective integration of PEI and DOPA. SEM and TEM were used to examine the morphology, showing that AuNPs were evenly distributed inside the pores and on the membrane surface. Water permeation tests indicated that membrane modifications reduced pore size and flux, which is advantageous for regulating residence time in catalytic reactions. The DOPA-AuNP-modified membranes demonstrated high catalytic efficiency and structural integrity during continuous use, highlighting their potential for long-term environmental remediation.

In catalysis, the catalytic activity of nanoparticles can be optimized by functionalizing them with specific chemical groups tailored for particular reactions. Langer et al. investigated the effect of cationic polymer coatings on the catalytic activity of AuNPs [108]. Their study demonstrated that coatings with cationic polyelectrolytes, such as poly(allylamine hydrochloride) (PAH), significantly enhanced the catalytic activity of partially embedded gold nanoislands (AuNIs) in transfer hydrogenation and oxidation reactions involving anionic substrates. The polymer coatings were prepared by incubating AuNIs in aqueous solutions of the polymers that can be considered as physisorption of the polymers on the metal surface, allowing for adsorption and the formation of a positively charged layer [47,48,49,131]. The improved catalytic activity was attributed to a reduction in activation energy, facilitated by several possible mechanisms. These include electrostatic interactions that increase the local concentration of negatively charged reactants around catalytic sites, the generation of strong electric fields via image charges, and modifications to reactant binding strength that promote reaction kinetics. To ensure consistency and understand the effects of the polymer coatings, the AuNI slides were characterized using multiple techniques. SEM verified that the coatings did not alter the physical structure of the AuNIs. XPS confirmed the chemical composition and successful attachment of PAH to the gold surface. Additionally, LSPR spectroscopy was used to verify polymer attachment, as indicated by a red shift in the LSPR peak. This shift, observed via UV–Vis spectroscopy, resulted from the increased refractive index due to the polymer layer, confirming successful binding. This work underscores the potential of engineering the microenvironment of heterogeneous catalysts to enhance their efficiency, paving the way for hybrid materials that cooperatively interact with reactants.

These examples demonstrate how polymer coatings can expand AuNP functionality beyond biomedical and sensing contexts by tailoring surface charge, wettability, and diffusion control for catalytic and environmental systems. In remediation membranes, redox-active and adhesive polymers such as PDA and PEI promote in situ AuNP immobilization, yielding mechanically robust, reusable materials for continuous pollutant degradation. Conversely, cationic coatings like PAH enhance catalytic turnover by electrostatically attracting anionic substrates and tuning reaction energetics near the surface. Compared with drug-delivery and biosensing systems, where polymer shells must balance biocompatibility and responsiveness, catalytic and environmental coatings favor charge-assisted reactivity and long-term stability under harsher conditions. These distinctions highlight that optimizing polymer-substrate compatibility and interfacial charge distribution is central to achieving durable performance in polymer-coated AuNP systems for environmental and catalytic use [107,108].

#### 3.1.4. Polymer-Coated AuNPs in Electronics

**Figure 6:**
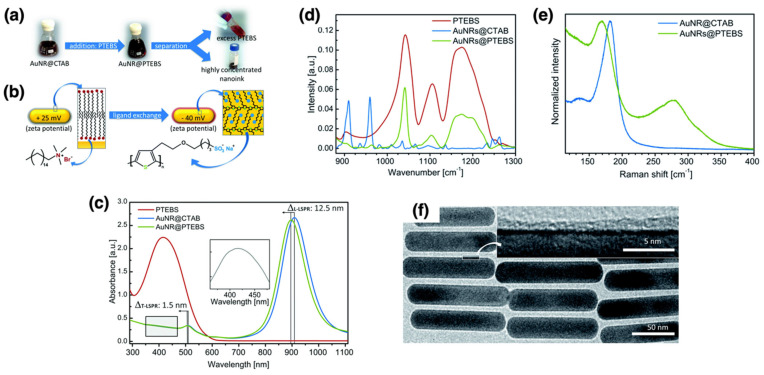
(**a**) Images of dispersions taken before and after ligand exchange. (**b**) Schematic representation of the surface chemistry of AuNRs before and after ligand exchange. (**c**) UV–Vis/NIR spectra of AuNR@CTAB, AuNR@PTEBS, and pure PTEBS. Inset: Comparison of the UV–Vis spectra of AuNR@PTEBS and AuNR@CTAB. (**d**) IR spectra of PTEBS, AuNR@CTAB, and AuNR@PTEBS. (**e**) Raman spectra of AuNR@CTAB and AuNR@PTEBS. (**f**) TEM images of AuNR@PTEBS at low and high magnification. Reproduced with permission from Ref. [109]. Copyright 2016 Royal Society of Chemistry.

Functionalized polymer layers can enhance the performance of conductive inks and coatings, improving their durability and conductivity in electronic applications. Reiser et al. developed sintering-free conductive inks using gold nanorods (AuNRs) coated with conjugated polymers, specifically poly[2-(3-thienyl)-ethyloxy-4-butylsulfonate] (PTEBS) [109]. The coating method can be considered a “grafting to” approach due to the bonding between the pre-formed polymers and AuNRs [51,52,54]. Nanorods were preferred over spherical nanoparticles due to their higher aspect ratio and anisotropic properties, which enhance electron transport and lower the percolation threshold. The ligand exchange method with PTEBS enhanced the colloidal stability and electrical conductivity of AuNRs, which were confirmed using zeta potential measurements, UV–Vis/NIR spectroscopy, infrared (IR) spectroscopy, Raman spectroscopy, and TEM (Figure 6a–f). The surface charge of the AuNRs was evaluated using zeta potential measurements before and after ligand exchange. Initially, cetyltrimethylammonium bromide (CTAB)-coated AuNRs exhibited a positive zeta potential of +25 mV, indicating the presence of a cationic surfactant. After ligand exchange with PTEBS, the zeta potential shifted to −40 mV, signifying successful replacement of CTAB by the negatively charged PTEBS polymer. This change in surface chemistry enhanced the colloidal stability of AuNRs in polar solvents. The LSPR peaks of the nanorods, which are sensitive to changes in the local dielectric environment surrounding the AuNRs, were observed using UV–Vis/NIR spectroscopy. Both the transverse (T-LSPR) and longitudinal (L-LSPR) peaks exhibited a blue shift after ligand exchange. The L-LSPR peak shifted to a shorter wavelength of 909 nm, indicating the successful replacement of CTAB with PTEBS polymer. This shift was attributed to interactions between the π-electrons in the conjugated polymer and the conduction band electrons of gold. To verify the chemical composition of the surface ligands on the AuNRs, IR spectroscopy was employed. The spectra displayed distinctive vibration bands of the PTEBS sulfonate groups, while CTAB-associated peaks disappeared, indicating complete ligand exchange. Raman spectroscopy further confirmed this exchange, revealing new peaks corresponding to Au-S bonds and the disappearance of the Au-Br bond signal, which is characteristic of CTAB-coated AuNRs. These findings suggest that the PTEBS polymer binds to the AuNRs via its thiophene rings, ensuring close contact between the conjugated polymer backbone and the gold surface. This conjugation facilitates electron transport between nanorods. The morphology of the AuNRs post-coating was examined using TEM. The consistent size and shape of the nanorods after polymer modification indicate that the polymer coating did not significantly alter their structural integrity, which is crucial for preserving their optical and electrical characteristics. The modified AuNRs formed stable, conductive inks that did not require post-deposition sintering. These sintering-free inks exhibited an electrical resistivity of approximately 7 × 10^−6^ Ω·m immediately after deposition, outperforming conventional nanoparticle-based inks that rely on thermal or photonic sintering. A related formulation incorporating PEDOT:PSS instead of PTEBS yielded films with even lower resistivity (9.9 × 10^−7^ Ω·m) and retained their performance for at least one year under ambient storage conditions, demonstrating long-term stability and suitability for flexible printed electronic applications. This property makes them suitable for flexible substrates and roll-to-roll printing processes. The inks exhibited significant conductivity immediately after deposition, outperforming conventional metal inks. The approach was also successfully applied to another polymer, poly(3,4-ethylenedioxythiophene) polystyrenesulfonate (PEDOT:PSS), yielding even higher conductivities. This study presents a scalable, sintering-free method for producing highly efficient conductive inks suitable for printed electronics while addressing key challenges such as post-deposition treatment requirements and compatibility with flexible substrates.

As a follow-up study, Klos et al. recently reported mechanically robust, inkjet-printable polymer nanocomposites incorporating PEDOT:PSS-coated hybrid AuNPs, referred to as hybrid conductive nanoparticles (hCNPs), combined with poly(vinyl alcohol) (PVA) [110].

As a follow-up study, Klos et al. reported mechanically robust, inkjet-printable nanocomposites composed of PEDOT:PSS-coated hybrid AuNPs combined with poly(vinyl alcohol) (PVA) [110]. The coating was achieved through a “grafting to” ligand exchange process [51,52,109]. The printed films exhibited conductivities of up to 2.1 × 10^5^ S·m^−1^ at 10 vol% PVA, and maintained resistivities of 9.5–13.1 μΩ·m over 280 days with minimal degradation. Incorporating PVA improved film adhesion to flexible substrates; films prepared without PVA delaminated during tape adhesion testing, while those containing PVA remained intact. SAXS measurements indicated reduced interparticle spacing with PVA addition, supporting the formation of continuous conductive pathways. The inks exhibited viscosities of 1.6–2.0 Pa·s and produced flexible printed tracks that retained their conductivity under mechanical deformation.

These results indicate that polymer-coated AuNP systems designed for printed electronics require a careful balance between conductive nanoparticle connectivity and polymer binder content. Conductive shells such as PEDOT:PSS facilitate efficient charge transport, while flexible binders such as PVA improve film integrity and adhesion without significantly diminishing conductivity when incorporated in controlled amounts. In contrast to biomedical or sensing applications—where polymer layers are optimized for biocompatibility, surface recognition, or responsiveness—electronic applications demand uninterrupted conductive pathways, low interfacial resistance, and uniform nanoscale morphology. Maintaining continuous conductive networks while preserving mechanical flexibility is therefore essential for achieving long-term electrical stability in polymer-coated AuNP-based printed electronic materials [109,110].

In summary, the multifunctional performance of polymer-coated AuNPs, enabled by various types of polymer layers, makes them highly versatile and valuable for a wide range of advanced applications. Their ability to enhance electrical, mechanical, and environmental properties continues to drive innovation across multiple scientific, technological, and biomedical fields.

### 3.2. Silver Nanoparticles (AgNPs)

Silver nanoparticles (AgNPs) exhibit several exceptional properties that make them highly valuable in nanotechnology and a wide range of applications. One of the most notable properties of AgNPs is their extraordinary antimicrobial activity, which is significantly stronger than that of many other metal nanoparticles. This broad-spectrum antimicrobial effect is primarily attributed to the release of silver ions, which interact with microbial cell membranes, proteins, and DNA, resulting in cell death. This property is particularly advantageous in medical and environmental applications where pathogen control is essential [132,133,134].

AgNPs also possess unique optical properties, characterized by strong absorption and scattering of light due to their LSPR. This phenomenon enhances sensitivity in optical applications such as imaging, biosensing, and photothermal therapy [134]. Additionally, AgNPs exhibit excellent electrical conductivity, making them highly suitable for use in electronic applications, including conductive inks and flexible electronics [135]. Furthermore, AgNPs demonstrate notable thermal stability, allowing them to maintain their structural integrity and functional properties at elevated temperatures, which is favorable in various industrial processes [136].

When coated with polymer, AgNPs acquire additional functionalities that extend their applicability to fields such as antimicrobial treatments, drug delivery, biosensing, environmental applications, and electronics. The following sections discuss the enhanced properties and applications of polymer-coated AgNPs.

#### 3.2.1. Polymer-Coated AgNPs in Antimicrobials and Therapeutics

**Figure 7:**
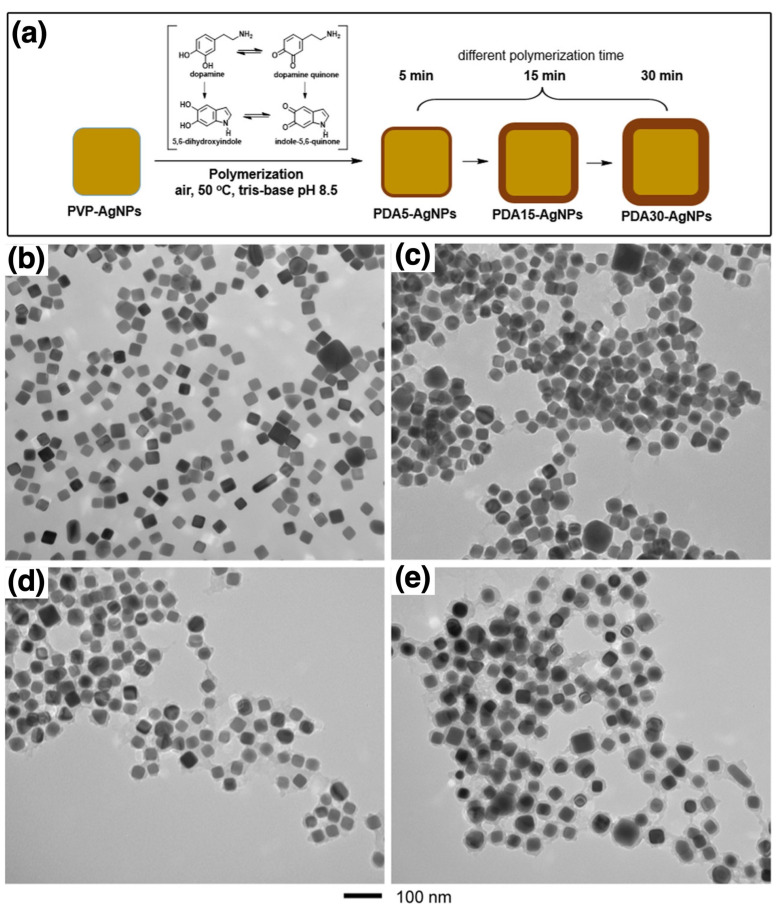
(**a**) Schematic representation of the PDA deposition process on PVP-AgNPs to form PDA-AgNPs at different polymerization times. (**b**–**e**) TEM images of AgNPs: (**b**) PVP-AgNPs; (**c**–**e**) PDA-AgNPs, also known as PDA5-AgNPs (**c**), PDA15-AgNPs (**d**), and PDA30-AgNPs (**e**), corresponding to PDA coating times of 5, 15, and 30 min, respectively. Reproduced with permission from Ref. [111]. Copyright 2020 American Chemical Society.

Polymer-coated AgNPs are of significant interest in various fields, particularly in medicine, where their potent antimicrobial properties are utilized for medical device coatings and therapeutic applications. Niyonshuti et al. investigated the synergistic antimicrobial effects of PDA surface coatings on AgNPs [111]. The study aimed to enhance the antimicrobial activity of AgNPs using PDA, which is known for its strong adhesive properties, rich chemical functionalities, and biocompatibility. Figure 7a illustrates the synthesis of PDA-coated AgNPs (PDA-AgNPs) by varying polymerization times to control the coating thickness from approximately 3 to 23 nm, as confirmed by TEM images (Figure 7b–e). Poly(vinylpyrrolidone)-coated AgNPs (PVP-AgNPs) were synthesized using the polyol method, a widely used technique for producing metal nanoparticles, particularly AgNPs. Once stabilized, dopamine hydrochloride was added to the solution of PVP-coated AgNPs, where it self-polymerized under basic conditions to form a PDA layer around the nanoparticles. The PDA coating was achieved through coordination between the Ag metal and the catechol groups of the in situ synthesized PDA polymers, which can be considered a “grafting to” method [51,52]. To characterize the PDA-AgNPs, UV–Vis spectroscopy was used to monitor LSPR peaks, which exhibited a red shift, confirming the successful deposition of PDA on the nanoparticle surfaces. Thick PDA coatings resulted in a more pronounced LSPR shift. XPS further supported the presence of the PDA coating by revealing an increased nitrogen-to-silver (N/Ag) ratio, which correlated with the PDA layer thickness. Fourier transform infrared (FTIR) spectroscopy was used to confirm the presence of characteristic PDA functional groups, such as catechol, on the nanoparticle surface. To assess antimicrobial efficacy, a fluorescence-based bacterial growth test was conducted using *Escherichia coli* (*E. coli*). The results demonstrated that PDA-AgNPs exhibited significantly higher antimicrobial activity compared to PVP-AgNPs and PDA alone. The study indicated that the presence of a PDA coating enhanced the antimicrobial properties of AgNPs by increasing the production of reactive oxygen species (ROS) and facilitating interactions between AgNPs and the catechol groups of PDA. These findings highlight the potential of PDA-AgNPs as effective antimicrobial agents for various applications, including medical devices and healthcare products. The study underscores the significance of nanoparticle surface modifications in the development of advanced hybrid antimicrobial nanomaterials.

**Figure 8:**
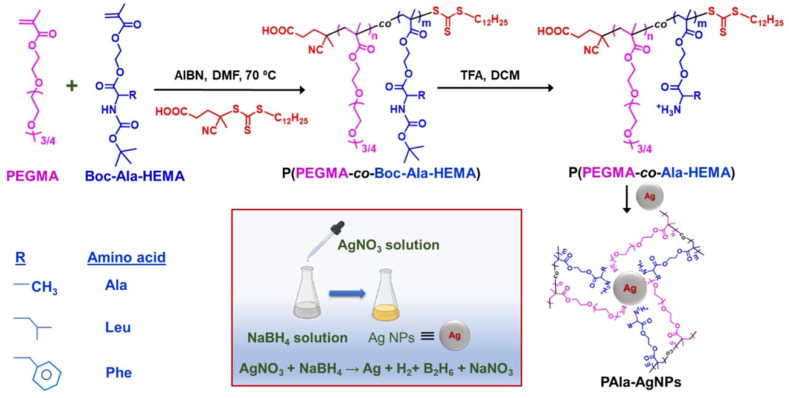
Schematic illustration of the synthesis of PC-AgNPs. Reproduced with permission from Ref. [112]. Copyright 2023 American Chemical Society.

Bera et al. investigated the inhibitory effects of amino acid-based polymer-coated AgNPs (PC-AgNPs) on insulin fibrillation [112]. They synthesized three copolymers using RAFT polymerization, incorporating poly(ethylene glycol) methyl ether methacrylate (PEGMA) and tert-butoxycarbonyl (Boc)-protected amino acids (alanine (Ala), leucine (Leu), and phenylalanine (Phe)). 2,2′-Azobisisobutyronitrile (AIBN) was used as the initiator, with thermal activation as the initiation method. The deprotected copolymers were then coated onto AgNPs via physisorption, likely due to the interfacial interaction between positively charged polymers and negatively charged nanoparticles [44,45,46]. An illustration of the reactions is shown in Figure 8. The PC-AgNPs were characterized using UV–Vis spectroscopy, DLS, TEM, and zeta potential measurements to confirm efficient polymer coating and nanoparticle stability. The study revealed that phenylalanine-based PC-AgNPs (PPhe-AgNPs) exhibited the highest inhibitory effect on insulin fibrillation, primarily due to hydrophobic interactions and surface charge effects. Further characterization, including Thioflavin T (ThT) fluorescence assays and TEM imaging, confirmed both the inhibition of insulin aggregation and the disintegration of matured fibrils. This study highlights the potential of amino acid-based PC-AgNPs as effective inhibitors of insulin fibrillation, suggesting promising therapeutic applications for amyloid-related conditions, such as type II diabetes.

Together, these studies demonstrate that the biological performance of AgNP-based nanomaterials is governed by the chemistry and structure of their polymer coatings. Dopamine-derived layers such as PDA promote strong bacterial inhibition through enhanced cell contact and reactive oxygen species generation, while amino acid-based copolymers fine-tune surface charge and hydrophobicity to suppress protein aggregation and amyloid formation. These contrasting strategies reveal how precise polymer design can direct a single metallic platform toward either antimicrobial or therapeutic functions. Compared with polymer-coated AuNPs that prioritize stability and targeted delivery, AgNP systems require stricter regulation of ion release and colloidal stability to reduce cytotoxicity. Fine control of coating thickness and surface composition is therefore essential for realizing safe and multifunctional AgNPs in biomedical applications [111,112].

#### 3.2.2. Polymer-Coated AgNPs in Targeted Drug Delivery and Biosensing

**Figure 9:**
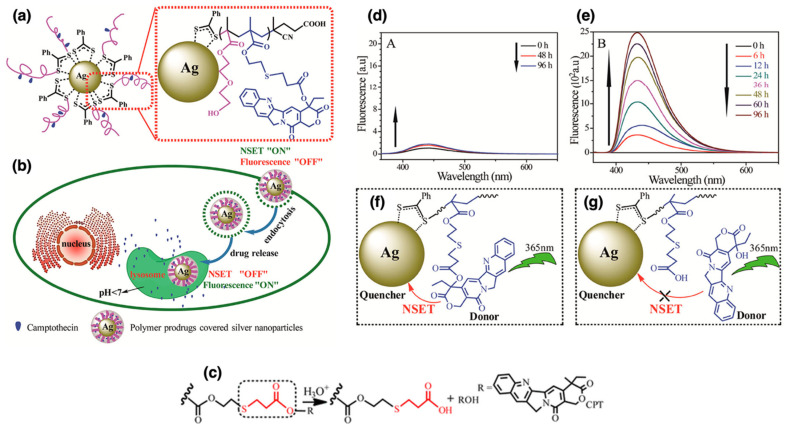
(**a**) Schematic illustration of the targeted drug-delivery system. (**b**) Intracellular release process of CPT. (**c**) Schematic representation of the β-thiopropionate bond cleavage in an acidic environment. The dashed box highlights the cleavable β-thiopropionate moiety. (**d**,**e**) Fluorescence spectra of the hybrid AgNPs after incubation in PBS solution at pH 7.4 and pH 5.0, respectively, over various time intervals. (**f**,**g**) Schematic diagrams of NSET “on” and NSET “off”, respectively, for P(HEO_2_MA-co-MACPT)@AgNPs. Reproduced with permission from Ref. [113]. Copyright 2017 American Chemical Society.

Polymer coatings enhance the stability and biocompatibility of AgNPs, making them suitable for targeted drug delivery and advanced biosensing applications. Qiu et al. developed a unique targeted drug-delivery system in which AgNPs were coated with camptothecin (CPT)-based polymer prodrugs, poly(2-(2-hydroxyethoxy)ethyl methacrylate-*co*-methacryloyloxy-3-thiahexanoyl-camptothecin) (P(HEO_2_MA-*co*-MACPT)) [113]. The polymer prodrug contained CPT attached via an acid-labile β-thiopropionate bond and was synthesized through RAFT polymerization using AIBN as the initiator and heat as the initiation method. The AgNPs were synthesized via the Lee-Meisel method, which involves the reduction of silver nitrate by sodium citrate, providing a cost-effective and scalable approach for producing stable AgNPs. Due to their antimicrobial properties, versatile functionalization, and biocompatibility, AgNPs were chosen as the nanocarrier in this study. These nanoparticles were then coated with the polymer prodrug via “grafting to” method, as confirmed by the formation of Ag–S chelate bonds between the pre-synthesized AgNPs and the polymers [51,52,137]. This system exhibited a switchable fluorescence “off” and “on” property due to nanoparticle surface energy transfer (NSET). Under neutral conditions, the CPT fluorescence was quenched (“off”). However, under acidic conditions, such as those found in tumor cells or within lysosomes, CPT was released, restoring its fluorescence (“on”), as shown in Figure 9a,b. The acid-labile β-thiopropionate bond played a critical role in this study, enabling controlled CPT release based on pH levels (Figure 9c). In the neutral pH of the bloodstream, the bond remained stable, preventing premature drug release. However, in the acidic environment of tumor cells, the bond cleaved, releasing CPT directly at the tumor site. This targeted and controlled drug-delivery mechanism improved therapeutic efficacy while minimizing potential side effects. The ability to switch fluorescence “on” and “off” allowed for real-time monitoring of drug release in living cells. As illustrated in Figure 9d–g, in vitro studies with HeLa cells confirmed successful CPT delivery, with fluorescence signaling drug release within lysosomes. This switchable mechanism offers precise control over drug release and enables real-time visualization of the process, ultimately improving the accuracy and efficiency of cancer treatment. The system holds promising implications for cancer therapy, providing a means to precisely control drug release and monitor therapeutic process in real-time.

In a recent study on polymer-coated AgNPs for biosensing applications, Kato et al. introduced an innovative single-step approach to synthesizing polymer-coated AgNPs with the goal to enhance fluorescence while effectively suppressing quenching [114]. By using poly-L-lysine (PLL) coupled with disulfide-containing PEGylated succinimidyl 3-(2-pyridyldithio)propionate (PEG4-SPDP), they successfully coated the nanoparticles through the “grafting to” method [51,52,138], forming a stable layer approximately 4 nm thick. This coating thickness was optimized to be thin enough to enable significant plasmonic fluorescence enhancement while thick enough to suppress nonradiative energy transfer, thereby reducing quenching. TEM imaging confirmed the core–shell structure, while zeta potential measurements indicated excellent stability of the coated nanoparticles. The study demonstrated a 26-fold fluorescence enhancement by inhibiting nonradiative energy transfer, which was verified by both experimental analysis and numerical simulations. The disulfide bonds within PEG4-SPDP cleaved in the presence of silver, forming durable covalent bonds that ensured long-term stability. Additionally, PLL provided versatility for surface modification, allowing conjugation with different biomolecules and dyes to enhance the functionality of the nanoparticles. This novel approach presents significant potential for highly sensitive biosensing and bioimaging applications, including live-cell imaging, by maximizing fluorescence enhancement and minimizing quenching.

Recent advances in polymer-coated AgNPs underscore the critical influence of surface chemistry on their functional performance in biomedical contexts. Polymer prodrug systems incorporating CPT enable pH-responsive drug release and in situ fluorescence tracking, while PLL and PEG-based architectures promote enhanced optical response and colloidal stability for biosensing and imaging applications. In comparison with PDA- and amino acid-modified AgNPs, which primarily target antimicrobial efficacy and protein aggregation control, these approaches emphasize tunable optical behavior and stimulus sensitivity. Precise control of polymer thickness, interfacial bonding, and physicochemical stability therefore remains central to the rational design of multifunctional AgNPs for therapeutic and diagnostic implementation [111,112,113,114].

#### 3.2.3. Polymer-Coated AgNPs in Environmental Applications

In environmental science, polymer-coated AgNPs are utilized for water purification and as antimicrobial surface coatings to prevent contamination. Skiba et al. synthesized PVP-coated AgNPs using contact non-equilibrium low-temperature plasma (CNP) and examined their application in water purification [115]. The CNP technique involves generating a plasma discharge at the gas–liquid interface, where reactive species produced in the plasma effectively reduce silver ions from silver nitrate, forming AgNPs via an in situ approach [68,139,140]. By adjusting PVP levels, the effectiveness, stability, and size of the AgNPs were analyzed. The CNP process generated active species that efficiently reduced silver ions into metal nanoparticles, resulting in the formation of consistently distributed AgNPs. Additionally, the CNP method promoted strong interactions between AgNPs and PVP, effectively stabilizing the nanoparticles and preventing aggregation. Various characterization techniques, including UV–Vis spectroscopy, TEM, X-ray diffraction (XRD), and zeta potential analysis, confirmed the antibacterial effectiveness of PVP-coated AgNPs against *E. coli* and *Staphylococcus aureus* (*S. aureus*). Moreover, pre-synthesized PVP-coated AgNPs were incorporated into alginate, a biopolymer known for its biocompatibility and gel-forming characteristics, to form composite beads via ionotropic gelation. These beads were tested in column filtration experiments, demonstrating efficient removal of *E. coli*-contaminated water. The study showed that CNP-produced PVP-coated AgNPs serve as effective antibacterial agents for water purification. Both PVP concentration and CNP processing conditions were identified as key factors influencing nanoparticle performance.

This work highlights the effectiveness of PVP-coated AgNPs as stable and efficient antibacterial agents for environmental purification. The CNP synthesis method enabled uniform particle formation, while alginate incorporation improved practical applicability in water treatment systems.

#### 3.2.4. Polymer-Coated AgNPs in Electronics

Polymer-coated AgNPs play a crucial role in the development of conductive inks and nanocomposites for flexible and printed electronics, demonstrating their adaptability in next-generation electronic devices. Khalil et al. fabricated nanocomposite films containing nanofibrillated cellulose (NFC), PVP, and AgNPs to evaluate their mechanical and electrical conductivity properties [116]. NFC was extracted from rice straw pulp, while AgNPs were synthesized in situ [68,139,140] within the PVP matrix by reducing silver nitrate directly in PVP. This process ensured uniform dispersion and strong polymer-nanoparticle interactions, improving electrical conductivity and mechanical integrity. The NFC/PVP/AgNP nanocomposites exhibited flexibility and tensile strength, making them suitable for wearable electronics. The inclusion of AgNPs significantly enhanced electrical conductivity by ensuring intimate contact between nanoparticles and the polymer matrix. Characterization techniques such as UV–Vis spectroscopy, TEM, XRD, SEM, and energy dispersive X-ray spectroscopy (EDX) confirmed the homogeneous distribution of AgNPs within the PVP matrix, further supporting their tensile properties and enhanced conductivity by tensile strength testing and high-resolution broad band impedance analyzer, respectively. The study suggested that these nanocomposite films could be applied in sensitive electronic packaging, highlighting the versatility of polymer-coated AgNPs in electronic applications.

The integration of polymer-coated AgNPs into electronic materials demonstrates their versatility beyond biomedical and environmental applications. Using a PVP matrix enabled uniform nanoparticle dispersion and efficient charge transport, resulting in flexible, conductive, and mechanically stable nanocomposite films. This study highlights the significance of controlled polymer-nanoparticle interactions in enhancing the performance of AgNPs for modern electronic technologies.

### 3.3. Copper Nanoparticles (CuNPs)

Copper nanoparticles (CuNPs) have generated significant interest due to their unique characteristics and broad range of applications. These nanoparticles exhibit excellent electrical and thermal conductivity, catalytic activity, and antimicrobial properties, making them valuable in various scientific and industrial fields. Due to their high electrical conductivity, CuNPs are particularly suitable for applications in electronics and conductive materials [141]. Their catalytic properties are leveraged in chemical reactions such as hydrogenation and oxidation, as they reduce activation energies and enhance reaction rates [142]. Moreover, CuNPs demonstrate substantial antimicrobial activity, proving effective against various pathogens including bacteria and fungi, thereby extending their utility in medical applications [143]. In electronics, CuNPs are utilized in conductive inks and flexible electronic devices. Their high thermal conductivity also makes them well-suited for heat sinks and thermal interface materials [144,145].

Polymer-coated CuNPs have emerged as a promising avenue for enhancing their stability and expanding their potential applications, particularly in drug delivery and antimicrobials. The following sections explore these emerging applications.

#### 3.3.1. Polymer-Coated CuNPs in Drug Delivery

**Figure 10:**
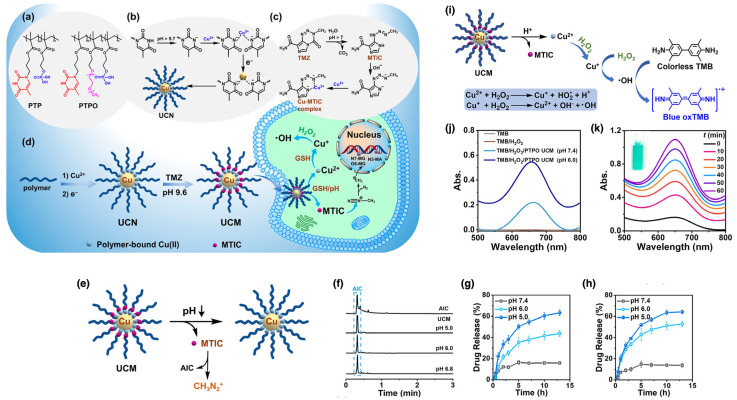
(**a**) Chemical structures of PTP and PTPO polymers. (**b**–**d**) Schematic representation of polymer–copper coordination, copper–MTIC coordination, the preparation of polymer-templated UCMs, and the dual-responsive release of MTIC and copper in response to GSH and pH for DNA methylation and catalytic hydroxyl radical (•OH) generation in tumor cells. (**e**) Schematic diagram illustrating the release of MTIC from UCMs under acidic conditions, leading to the production of CH_3_N_2_^+^. (**f**) UPLC analysis using a PDA detector (λ = 270 nm) to assess the filtrate of PTP UCM at different pH levels. (**g**,**h**) Cumulative drug release profiles of PTP UCM and PTPO UCM, respectively, following incubation at 37 °C under various pH conditions. (**i**) Schematic representation of the Fenton reaction catalyzed by copper, the colorimetric detection of •OH, and the stimuli-induced degradation of UCMs. (**j**) UV–Vis spectra of TMB alone and after incubation for 20 min with various reactive species. (**k**) Time-dependent UV–Vis spectra of TMB incubated with PTPO UCM at the pH 6.0. Reproduced with permission from Ref. [117]. Copyright 2021 American Chemical Society.

In the biomedical field, polymer-coated CuNPs have been investigated for their antimicrobial properties and potential in drug delivery systems. Wang et al. developed a biomimetic polymer-templated CuNP system to enhance the efficacy of temozolomide (TMZ) in treating glioblastoma multiforme (GBM) [117]. This system is designed to respond to the pH and glutathione (GSH) concentrations in tumor microenvironments. GSH, a naturally occurring antioxidant that is typically present in elevated levels in tumor cells, interacts with CuNPs, breaking the copper–3-methyl-(triazen-1-yl)imidazole-4-carboxamide (MTIC) coordination and triggering the release of the stabilized MTIC, as illustrated in Figure 10a–d. It is essential to target cancer cells with this selective release mechanism in order to minimizing damage to healthy tissues.

The CuNPs and the final hybrid nanoparticles were synthesized in situ [68,146,147] using anionic copolymers, specifically poly(thymine-phosphate)methacrylate (PTP) and poly(thymine-phosphate-oligo(ethylene glycol)) (PTPO). These copolymers were produced through radical polymerization initiated by AIBN initiator in the presence of heat. These copolymers, collectively termed UCN, were functionalized with thymine groups to mimic DNA interactions, thereby stabilizing the primary TMZ intermediate, MTIC, forming the UCN-MTIC complex (UCM). This stabilization process prevents premature drug degradation and non-specific activation. Furthermore, CuNPs facilitate hydroxyl radical generation via a Fenton reaction, enabling a dual approach combining chemotherapy and chemodynamic therapy. The Fenton reaction, catalyzed by CuNPs, converts endogenous hydrogen peroxide into hydroxyl radicals, inducing oxidative stress and promoting cancer cell apoptosis.

This strategy significantly improved MTIC stability, resulting in only 17% degradation over 36 h. Moreover, the system exhibited enhanced cytotoxicity against both TMZ-sensitive and TMZ-resistant GBM cells while minimizing toxicity to normal cells. Several characterization techniques were employed to confirm system functionality. FTIR indicated copper-thymine coordination through weakened N-H stretching vibrations, while XPS confirmed the presence of both Cu(0) and Cu(II) in UCNs. UV–Vis spectroscopy revealed red-shifted absorption peaks after MTIC immobilization, indicating successful encapsulation and stabilization. Ultrahigh-performance liquid chromatography (UPLC) was employed to quantify MTIC degradation products (AIC and CH_3_N_2_^+^, depicted in Figure 10e). The release kinetics of the MTIC under varying pH conditions was also examined with the help of a dialysis method, as illustrated in Figure 10f–h. The generation of hydroxyl radicals was validated using 3,3′,5,5′-tetramethylbenzidine (TMB) as a specific probe. The oxidation of TMB into a blue-colored species (oxTMB) with a maximum absorption at 657 nm, as illustrated in Figure 10i–k, confirmed the successful development of a dual-function therapeutic system. This system not only improved MTIC stability but also demonstrated heightened cytotoxicity against both TMZ-sensitive and TMZ-resistant GBM cells while exhibiting reduced toxicity to healthy cells. The dual-action strategy, integrating chemotherapy with chemodynamic therapy, represents a promising approach for enhancing TMZ stability and efficacy in GBM treatment, marking a significant advancement in cancer therapy. The incorporation of thymine groups to mimic DNA interactions played a pivotal role in the system’s design, significantly improving drug stabilization and delivery performance.

This work illustrates how polymer-functionalized CuNPs can serve as multifunctional therapeutic platforms by integrating chemical reactivity with controlled drug delivery. Unlike AuNP- and AgNP-based carriers, which rely mainly on surface functionalization and responsive polymer shells for release control, CuNPs introduce an intrinsic redox capability that enables combined chemotherapy and chemodynamic therapy. The thymine phosphate copolymer template not only stabilizes the active MTIC intermediate but also supports selective drug release in response to tumor-specific pH and glutathione levels, offering a targeted and oxidative stress-enhanced treatment strategy. Compared with noble metal systems, this approach provides both higher therapeutic efficiency and cost-effectiveness, while maintaining biocompatibility through rational polymer coordination chemistry. These findings highlight the unique potential of polymer-coated CuNPs for developing dual-function anticancer nanotherapeutics.

#### 3.3.2. Polymer-Coated CuNPs in Antimicrobials and Environmental Applications

The catalytic properties of CuNPs are widely utilized in antimicrobials, where they facilitate the degradation of microbial cells. Bogdanović et al. investigated the formation and antimicrobial properties of a copper-polyaniline (Cu-PANI) nanocomposite [118]. The Cu-PANI nanocomposite was synthesized via an in situ oxidative polymerization technique, where monomers polymerize in the presence of an oxidizing agent, leading to the simultaneous formation of CuNPs and polyaniline (PANI). CuNPs were selected for their potent and cost-effective antimicrobial properties, as well as their ability to interact with and disrupt microbial membranes. Moreover, the ability of copper to transition between cuprous (Cu(I)) and cupric (Cu(II)) oxidation states enhances its antimicrobial activity. PANI was chosen as the polymer matrix due to its environmental durability, reversible redox behavior, ease of production, cost-effectiveness, and inherent antibacterial properties. The dendritic structure of PANI also provides a high surface area, enhancing the interaction between the polymer matrix and the CuNPs. TEM analysis revealed that the CuNPs had an average diameter of 6.0 ± 0.2 nm and were homogenously dispersed within the composite. The antimicrobial efficacy of the Cu-PANI nanocomposite was assessed against *E. coli*, *S. aureus*, and *Candida albicans* (*C. albicans*), demonstrating significantly enhanced antimicrobial activity compared to CuNPs or PANI alone. Atomic force microscopy (AFM) analysis showed substantial structural damage to microbial cells upon exposure to the nanocomposite, leading to cell wall disruption and membrane collapse, which inhibited microbial growth. The study highlighted the synergistic effects of CuNPs and PANI, where PANI stabilized CuNPs by preventing aggregation and increasing their bioavailability. Moreover, the large surface area of PANI nanofibers facilitated interactions with microbial cell walls. The study concludes that the Cu-PANI nanocomposite is a highly effective and cost-efficient antimicrobial agent with potential applications in chemical and biological sensing.

Another notable example is the work of Duan et al., who developed halloysite nanotubes (HNTs) decorated with CuNPs within a mixed matrix membrane for water purification [119]. HNTs were selected due to their unique tubular structure, high surface area, porosity, chemical reactivity, mechanical stability, cost-effectiveness, and natural abundance. In this study, poly(4-vinylpyridine) (P4VP) was grafted onto HNTs through reverse atom transfer radical polymerization (RATRP), with AIBN as the initiator and CuCl_2_ as the catalyst. CuNPs were subsequently deposited onto the P4VP-grafted HNTs by reducing Cu^2+^ ions with sodium borohydride (NaBH_4_), forming CuNPs@HNTs via an in situ approach [68,148]. The final mixed matrix membranes were prepared by incorporating CuNPs@HNTs into a polyethersulfone (PES) matrix using the phase inversion method. Characterization using TEM, TGA, contact angle measurements, and AFM confirmed the successful integration of HNTs with CuNPs, leading to improved thermal stability, increased hydrophilicity, and smoother membrane surfaces. Antibacterial testing against *E. coli* revealed a high bacteriostasis rate of 94.5%, which caused bacterial membrane and DNA damage. Minimal copper leaching was observed, indicating the membranes’ suitability for water purification. The study concluded that these hybrid mixed matrix membranes exhibit enhanced water flux and strong antibacterial properties, underlining their potential for use in water treatment.

Therefore, both studies demonstrate how polymer coordination and composite structure influence the antimicrobial performance of CuNP-based systems. The Cu-PANI nanocomposite achieved strong, broad-spectrum antibacterial effects through synergistic redox activity and stabilization within a conductive polymer matrix, while the P4VP-grafted HNT membranes combined high surface area and structural stability with sustained antibacterial activity and minimal copper release. Together, these results highlight that optimizing polymer architecture and nanoparticle dispersion is key to achieving durable, efficient, and environmentally compatible CuNP-based antimicrobial materials [118,119].

### 3.4. Platinum Nanoparticles (PtNPs)

Platinum nanoparticles (PtNPs) have been extensively studied due to their exceptional properties and adaptability. As a noble metal, platinum resists oxidation and corrosion, ensuring long-term performance in various applications. The biocompatibility of PtNPs makes them particularly suitable for biomedical applications, including drug delivery and diagnostic imaging. Furthermore, PtNPs possess antioxidant properties that can mitigate ROS generation during photothermal cancer therapy [149,150]. Polymer coatings further enhance the stability and functionality of PtNPs, improving their potential applications in biomedical sensing and energy conversion [120,121].

#### 3.4.1. Polymer-Coated PtNPs in Biomedical Sensing

Polymer-coated PtNPs combine the unique properties of PtNPs with the functional advantages of polymers, enhancing durability, dispersion, and biocompatibility. The polymer layer prevents nanoparticle aggregation, ensuring stability across different environments. Furthermore, polymer coatings can be tailored to include targeting agents for medical applications or functional groups that enhance catalytic activity.

As one of the most recent and noteworthy studies in the biomedical field, Deng et al. reported the synthesis and analysis of a chitosan-encapsulated platinum nanoparticle (Ch-PtNP) hybrid nanocomposite that demonstrates notable oxidase-like activity suitable for sensing applications [120]. Chitosan was selected for its biocompatibility, non-toxicity, and functional groups that interact with metal ions. The synthesis process involved a single-step borohydride reduction method of PtCl_6_^2−^, which can also be considered an in situ approach [68,71], with chitosan serving as both a stabilizer and a protective agent. Characterization using FTIR, XRD, and EDX confirmed the stable coordination between chitosan and PtNPs. The Ch-PtNPs catalyzed the oxidation of 3,3′,5,5′-tetramethylbenzidine (TMB) in the presence of dissolved oxygen, enabling colorimetric detection of acid phosphatase (ACP). Since ACP serves as a biomarker for diseases such as prostate cancer, bone disease, and lysosomal storage disorders, its detection is clinically significant. Monitoring ACP levels is crucial for early disease detection, tracking disease progression, and understanding enzyme function. The Ch-PtNPs exhibited selective detection abilities of ACP, with a limit of detection (LOD) of 0.016 U·L^−1^ and a linear range of 0.25–2.5 U·L^−1^, highlighting their potential for clinical diagnosis.

The synthesis of chitosan-stabilized PtNPs represents an efficient approach for developing biocompatible and sensitive biosensors. The resulting system exhibited excellent selectivity, stability, and a low detection limit, demonstrating how polymer coordination can improve catalytic efficiency and expand the clinical potential of PtNP-based sensing materials.

#### 3.4.2. Polymer-Coated PtNPs in Energy Conversion

**Figure 11:**
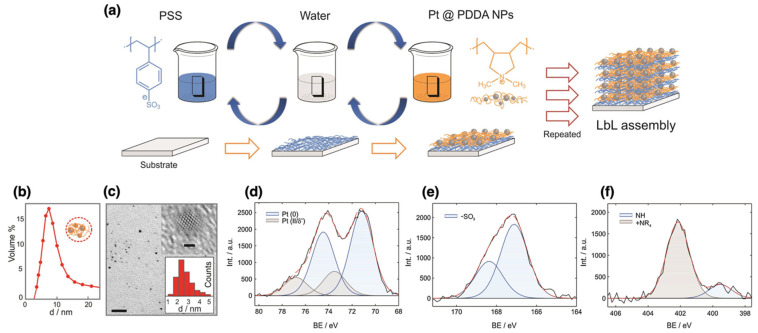
(**a**) Schematic representation of the layer-by-layer (LbL) assembly process. (**b**) DLS volume distribution of Pt@PDDA nanoparticles. (**c**) TEM image (scale bar: 20 nm) and HRTEM image (scale bar: 1 nm) of the nanoparticles, along with the particle size distribution histogram. (**d**–**f**) XPS spectra and corresponding peak fittings for the Pt 4f, S 2p and N 1s core-level regions of a 20-bilayer assembly, respectively. Reproduced with permission from Ref. [121]. Copyright 2017 Elsevier.

Polymer-coated PtNPs are highly efficient in energy conversion applications due to their enhanced stability and combined catalytic properties. Fenoy et al. developed a simple one-step method to synthesize polyelectrolyte-capped PtNPs for electrocatalysis in the hydrogen evolution reaction (HER) [121]. The PtNPs were produced by mixing poly(diallyldimethylammonium chloride) (PDDA) with hydrogen hexachloroplatinate (IV), followed by reduction with NaBH_4_. PDDA served as both a stabilizer and a capping agent, preventing nanoparticle aggregation and improving stability. The PtNPs were subsequently assembled into LbL structures using poly(sodium 4-styrenesulfonate) (PSS) as the counter-polyelectrolyte, as illustrated in Figure 11a. PSS can bind with PDDA through strong electrostatic interactions. It also offers stability, solubility, the ability to form uniform films, chemical compatibility, and enhancement of electrochemical properties. UV–Vis spectroscopy confirmed the reduction of platinum ions and the formation of PtNPs, while DLS and TEM, including high-resolution TEM (HRTEM), verified the size distribution and morphology. The analysis further revealed that the Pt@PDDA nanoparticles had an average diameter of 11 nm, with well-dispersed metallic cores measuring approximately 2.6 ± 0.6 nm (Figure 11b,c). AFM validated the homogeneity and thickness of the LbL assemblies, while attenuated total reflectance FTIR (ATR-FTIR) spectroscopy and XPS confirmed the chemical composition and oxidation states of elements in the films, specifically identifying the existence of sulfonate groups from PSS and nitrogen species from PDDA. The corresponding XPS data is presented in Figure 11d–f. The LbL assembly of PDDA-capped PtNPs offers a reliable and efficient method for producing electroactive coatings with excellent electrocatalytic performance for hydrogen evolution. This approach is straightforward, cost-effective, and results in films with outstanding electrochemical connectivity, rendering them highly suitable for various energy conversion applications.

This study exemplifies how polymer engineering can tailor PtNP performance for energy conversion applications. In contrast to biocompatible chitosan-based PtNPs developed for biomedical sensing, PDDA and PSS capped nanoparticles focus on electrochemical stability and catalytic efficiency. The combination of polyelectrolyte capping and layer-by-layer assembly produces uniform conductive films with strong hydrogen evolution activity and long term durability. These results illustrate how polymer choice determines functionality, enhancing biocompatibility in biomedical systems or improving charge transfer and structural stability in energy related technologies [120,121].

### 3.5. Palladium Nanoparticles (PdNPs)

Palladium nanoparticles (PdNPs) are a remarkable type of metal nanoparticles recognized for their outstanding catalytic properties, excellent electrical conductivity, and high stability [151]. They have been widely utilized in various applications, including catalytic converters, hydrogen storage, and organic synthesis. PdNPs are particularly valued in organic chemistry for their role in facilitating carbon-–carbon coupling reactions, such as Suzuki and Heck reactions [152,153]. Additionally, their exceptional electrical conductivity and stability in diverse environments make them highly suitable for use in electronic devices and sensors [154].

Despite these benefits, PdNPs, like other metal nanoparticles, often face challenges such as aggregation and potential toxicity, which can limit their efficiency for certain applications. One common approach to overcoming these limitations is the use of polymer coating, which can improve stability and biocompatibility. The following sections discuss the potential applications of polymer-coated PdNPs, particularly in drug delivery and sensor technologies.

#### 3.5.1. Polymer-Coated PdNPs in Drug Delivery

Polymer-coated PdNPs play a crucial role in drug delivery systems, where they facilitate the precise delivery of therapeutic agents to specific targets, thereby minimizing adverse effects and enhancing treatment efficacy. Polymer-coated PdNPs also contribute to diagnostic imaging and photothermal therapy, as their enhanced stability and compatibility improve both effectiveness and safety.

Bharathiraja et al. developed chitosan oligosaccharide (COS)-coated PdNPs functionalized with the RGD peptide (Pd@COS-RGD) to enable tumor targeting via multiple modalities, including imaging and therapy using light [122]. Palladium was selected for its stability, catalytic properties, and plasmonic behavior in the NIR range, offering an alternative to gold-based nanomaterials for photothermal therapy (PTT). The PdNPs were synthesized using a seed-mediated growth method with cetyltrimethylammonium chloride (CTAC) as a stabilizing agent. COS was modified with thioglycolic acid to introduce thiol groups, enabling ligand exchange on the PdNPs, which can be considered a “grafting to” method [51,52,155]. This modification involved the formation of amide bonds between the amine groups of COS and the carboxyl group of thioglycolic acid, facilitated by 1-ethyl-3-(3-dimethylaminopropyl) carbodiimide (EDC) as a catalyst. The COS-coated PdNPs were further functionalized with the RGD peptide using “thiol-ene click chemistry”, enhancing the nanoparticles’ ability to target tumors. Characterization techniques, including UV–Vis spectroscopy, TEM, XRD, FTIR, and TGA, confirmed the crystalline structure, uniform size distribution, and successful surface modifications of the PdNPs. The Pd@COS-RGD nanoparticles exhibited strong photothermal properties, generating heat upon exposure to an 808 nm laser, which was sufficient to induce cancer cell death. Additionally, they demonstrated robust photoacoustic signal generation, facilitating non-invasive tumor imaging. Drug release studies indicated that the nanoparticles exhibited pH-dependent release kinetics, with faster drug release occurring in acidic environments typical of cancer cells. This behavior was attributed to the rapid degradation of specific linkages within the nanocomposite under acidic conditions. In vitro cellular uptake studies demonstrated a high accumulation of Pd@COS-RGD in MDA-MB-231 breast cancer cells, leading to significant photothermal cytotoxicity under NIR laser exposure. In vivo experiments conducted in mice with MDA-MB-231 tumor cells further confirmed the preferential accumulation of Pd@COS-RGD in tumor tissues, with laser treatment resulting in substantial tumor suppression without observable toxicity or adverse effects.

Among the polymer coated noble metal nanoparticles discussed, PdNPs stand out for their multifunctionality and chemical robustness. Their superior photothermal conversion and oxidation resistance ensure consistent performance where CuNPs often face instability or ion leaching. The COS coating enhances dispersion and biocompatibility, while RGD functionalization enables selective tumor targeting. Moreover, PdNPs generate localized heat more efficiently than AuNPs, achieving higher ablation effects with reduced collateral damage [122,151,156]. In summary, this study highlights the successful development and application of PdNPs functionalized with COS and the RGD peptide, demonstrating enhanced stability, biocompatibility, and diverse therapeutic potential, particularly in targeted drug delivery and cancer treatment.

#### 3.5.2. Polymer-Coated PdNPs in Sensors

**Figure 12:**
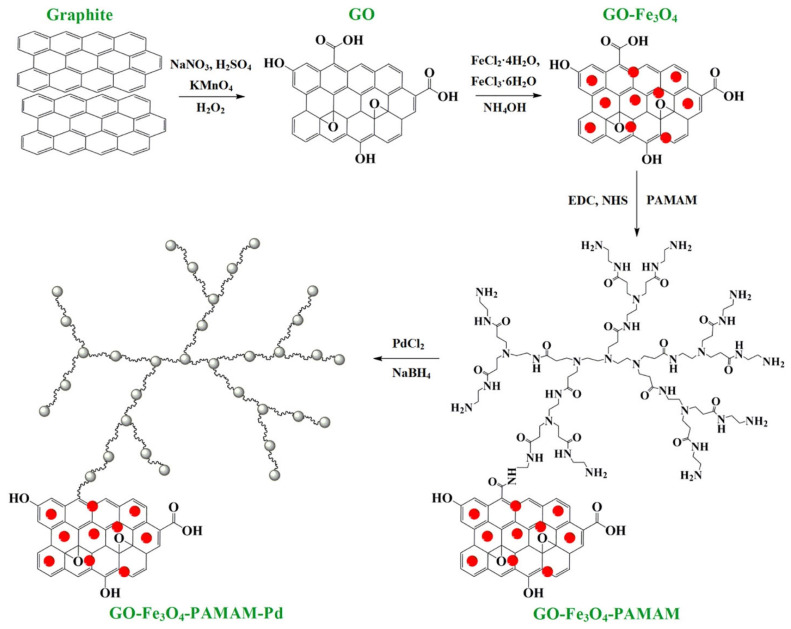
Synthesis of the GO-Fe_3_O_4_-PAMAM-Pd nanocomposite. Reproduced with permission from Ref. [123]. Copyright 2019 Elsevier.

Polymer-coated PdNPs are also extensively employed in advanced sensor technologies, where the polymer layer can be engineered to interact selectively with specific analytes. Baghayeri et al. developed a non-enzymatic hydrogen peroxide (H_2_O_2_) sensor based on dendrimer-functionalized magnetic graphene oxide decorated with PdNPs (GO-Fe_3_O_4_-PAMAM-Pd) [123]. The detection of H_2_O_2_ is essential in various fields, including environmental monitoring, industrial processes, and clinical diagnostics, as it is a byproduct of biochemical reactions and signaling pathways. The GO-Fe_3_O_4_-PAMAM-Pd nanocomposite was synthesized through a multiple–step process. First, magnetic graphene oxide (GO-Fe_3_O_4_) was prepared by co-precipitating Fe(II) and Fe(III) ions with ammonium hydroxide (NH_4_OH). The GO-Fe_3_O_4_ was then functionalized with an amine-terminated poly(amidoamine) (PAMAM) dendrimer using EDC and *N*-hydroxysuccinimide (NHS) as activation agents for the carbonyl groups on GO-Fe_3_O_4_, as illustrated in Figure 12. The PAMAM dendrimers were synthesized using the divergent method, an iterative polymerization approach initiated from an ethylenediamine (EDA) core. Each cycle involved Michael addition of methyl acrylate, followed by amidation with EDA, resulting in a highly branched, spherical structure. Finally, PdNPs were incorporated into the GO-Fe_3_O_4_-PAMAM composite using the incipient wetness impregnation method, wherein PdCl_2_ was reduced by NaBH_4_ through a process that enabled an in situ approach [68,157]. Graphene oxide was chosen for its large surface area, excellent conductivity, and applicability for surface modification, while Fe_3_O_4_ nanoparticles conferred magnetic properties for facile separation and enhanced conductivity. PAMAM dendrimers, known for their highly branched architecture and well-defined structure, provided numerous surface functional groups for PdNP anchoring, thus enhancing nanoparticle stability and dispersion. The unique dendritic structure facilitated uniform and dense PdNP decoration, ultimately improving the catalytic performance and stability of the nanocomposite. The GO-Fe_3_O_4_-PAMAM-Pd-modified glassy carbon electrode (GCE) demonstrated excellent electrocatalytic activity toward H_2_O_2_ reduction, with a broad linear detection range (0.05–160 μM) and a low LOD (0.01 μM). The sensor also exhibited high selectivity, strong anti-interference capability, and remarkable sensitivity. When tested in real water samples, it showed high recovery rates and reproducibility, confirming its potential for practical applications.

In summary, the dendrimer-stabilized PdNP hybrid nanomaterials demonstrate how controlled polymer architectures can support uniform nanoparticle distribution and enhance electrocatalytic performance. The resulting material provides high sensitivity, stability, and selectivity toward H_2_O_2_ detection, indicating strong potential for practical non-enzymatic sensing applications.

To summarize the design strategies and functional trends discussed for metal nanoparticles, Table 2 provides an overview of representative polymer-coated noble and base metal systems, including Au, Ag, Cu, Pt, and Pd nanoparticles. The table highlights their typical synthesis routes, polymer-coating chemistries, and application-specific parameters such as stability, shell thickness, and performance characteristics. This summary aims to facilitate direct comparison among different metal-polymer combinations and guide the rational design of nanostructures optimized for biomedical, catalytic, and sensing applications.

## 4. Structures, Properties, and Applications of Polymer-Coated Metal Oxide Nanoparticles

The integration of metal oxide nanoparticles with polymers marks a significant advancement in nanotechnology, combining the unique properties of inorganic cores with the versatility of organic shells. These nanoparticles are characterized by their distinctive chemical compositions, large surface areas, and diverse electrical, magnetic, and optical properties, all of which can be precisely tuned for a wide range of applications [9,10]. The polymer coating serves multiple functions, including preventing aggregation, improving dispersibility in various conditions, and enhancing biocompatibility. Additionally, functionalization of the polymer shell allows for the introduction of specific chemical groups, enabling the nanoparticles to respond to external stimuli such as pH, temperature, and magnetic fields, as well as facilitating interactions with biological molecules. Due to these attributes, polymer-coated metal oxide nanoparticles are widely applied in various fields such as environmental remediation, drug delivery, diagnostics, and catalysis [6,158].

The unique properties of these nanoparticles, including electrical conductivity, semiconducting behavior, chemical stability, magnetic characteristics, and photocatalytic activity, arise from their metal oxide cores. For instance, Fe_3_O_4_ nanoparticles (Fe_3_O_4_ NPs) are particularly valuable in applications such as magnetic resonance imaging (MRI), targeted drug delivery, and magnetic hyperthermia for cancer treatment due to their combination of electrical conductivity and superparamagnetic behavior. The electrical conductivity of Fe_3_O_4_ NPs, resulting from electron hopping between Fe^2+^ and Fe^3+^ ions, enhances their magnetic responsiveness and facilitates efficient energy transfer, making them especially useful in biomedical applications [159,160].

In contrast, ZnO and TiO_2_ exhibit semiconducting properties due to their wide bandgaps (3.0–3.2 eV for TiO_2_ and 3.37 eV for ZnO). Their ability to generate electron-hole pairs under UV light enables redox reactions that can degrade pollutants or drive chemical reactions such as water splitting in energy conversion systems. These photocatalytic properties are particularly valuable for environmental and energy-related applications [161,162,163]. Additionally, Al_2_O_3_ nanoparticles, especially in their α-phase, are renowned for their remarkable chemical and thermal stability, making them suitable for high-temperature and chemically resistant coatings [164].

The versatility of metal oxide nanoparticles in a wide range of cutting-edge applications is largely attributed to their ability to modify their electrical and semiconducting properties through surface modifications and polymer coatings. The following sections will provide an in-depth analysis of specific metal oxide nanoparticles, including Fe_3_O_4_, TiO_2_, ZnO, and Al_2_O_3_, detailing their compositions, characteristics, and applications. As summarized in Table 3, this discussion will explore the unique chemistry and interactions of these metal oxide nanoparticles with polymer coatings to highlight their potential for innovative applications in environmental science, energy conversion, and medicine.

### 4.1. Iron Oxide Nanoparticles (Fe_3_O_4_ NPs)

Iron oxide nanoparticles (Fe_3_O_4_ NPs) have attracted significant interest due to their unique physical and chemical properties, as well as their diverse applications. These nanoparticles exhibit strong magnetic properties, which can be tailored by adjusting their size, shape, and composition [159,160]. There are different forms of iron oxides, including magnetite (Fe_3_O_4_), maghemite (γ-Fe_2_O_3_), and hematite (α-Fe_2_O_3_), each possessing distinct characteristics that make them suitable for different applications. Fe_3_O_4_ is among the most commonly utilized iron oxides in nanoparticle technology due to its powerful magnetic properties.

When Fe_3_O_4_ NPs are reduced to a size below 20 nm, they exhibit superparamagnetism, meaning they become magnetized in the presence of an external magnetic field but do not retain magnetization once the field is removed. This characteristic prevents nanoparticle aggregation in the absence of a magnetic field, making them highly suitable for various biomedical applications such as MRI, targeted drug delivery, and hyperthermia treatment for cancer. Additionally, Fe_3_O_4_ possesses high saturation magnetization, making it suitable for applications requiring intense magnetic reactions [159,179,180].

Maghemite (γ-Fe_2_O_3_) is another form of iron oxide that shares similar magnetic properties with Fe_3_O_4_ but differs in its crystal structure and oxidation state. γ-Fe_2_O_3_ NPs also exhibit superparamagnetic behavior at small sizes, making them suitable for applications similar to Fe_3_O_4_. However, γ-Fe_2_O_3_ has a slightly lower saturation magnetization than Fe_3_O_4_ while still maintaining adequate magnetic properties for various applications [181].

Hematite (α-Fe_2_O_3_), another type of iron oxide, is recognized for its antiferromagnetic behavior at room temperature [182]. Although it does not exhibit superparamagnetism, α-Fe_2_O_3_ is widely utilized due to its chemical stability, non-toxicity, and availability. α-Fe_2_O_3_ NPs are employed in catalysis, as photocatalysts, and in sensor technologies [183,184].

The magnetic properties of Fe_3_O_4_ NPs can be customized by adjusting their size, shape, and surface coatings [159,185]. For instance, altering the particle size can shift their magnetic behavior from superparamagnetic to ferromagnetic or antiferromagnetic states [186]. Similarly, adjusting the nanoparticle shape can affect their coercivity and saturation magnetization, thereby optimizing their performance for specific uses [187]. In the ferromagnetic state, the magnetic moments of individual atoms or ions within a material align in the same direction under an external magnetic field, resulting in strong overall magnetization, which persists even after the field is removed—a phenomenon known as remanence. While materials like bulk iron and cobalt exhibit this behavior, certain iron oxide nanoparticles may also display ferromagnetic properties at the nanoscale, particularly when they exceed the superparamagnetic threshold. These properties are valuable in applications that require strong magnetic responses and retention. Conversely, in the antiferromagnetic state, the magnetic moments of atoms or ions align in opposite directions, canceling each other out and resulting in no net magnetization in the absence of an external magnetic field. This behavior is characteristic of materials such as α-Fe_2_O_3_ below the Morin transition (~260 K); at room temperature, α-Fe_2_O_3_ displays weak ferromagnetism due to spin canting [180,182,188].

Surface modification of Fe_3_O_4_ NPs with organic compounds and polymers further enhances their stability, dispersion, and biocompatibility, broadening their range of applications. The subsequent section discusses significant advancements in the development and application of polymer-coated Fe_3_O_4_ NPs, particularly in drug delivery, MRI, gene delivery, and hyperthermia treatment.

#### 4.1.1. Polymer-Coated Fe_3_O_4_ NPs in Drug Delivery

The functionalization of Fe_3_O_4_ NPs with biocompatible polymer coatings represents a significant advancement in nanomedicine. These coatings improve nanoparticle stability, enhance solubility in biological environments, and enable functionalization with targeting ligands, thereby improving drug delivery efficiency. The polymer layer acts as a protective barrier, minimizing potential toxicity and immune recognition, making Fe_3_O_4_ NPs more suitable for in vivo applications. Additionally, polymer coatings can be engineered to respond to external stimuli, such as pH or temperature changes, facilitating controlled drug release and enhancing therapeutic outcomes.

Sundaresan et al. described the synthesis of dual-responsive poly(*N*-isopropylacrylamide-acrylamide-chitosan) (PAC)-coated Fe_3_O_4_ magnetic nanoparticles (MNPs) designed for controlled and targeted drug delivery as well as imaging applications [165]. Fe_3_O_4_ NPs were synthesized via co-precipitation of Fe(II) and Fe(III) salts in the presence of NH_4_OH. To improve their compatibility with polymer matrices, the nanoparticles were functionalized with vinyltrimethoxysilane (VTMS). Silane-modified Fe_3_O_4_ NPs served as templates for constructing core–shell PAC-MNPs, possibly using the “grafting through” technique [51,189]. Free radical graft copolymerization was initiated in situ by the addition of tert-butyl hydroperoxide (TBHP) as the radical initiator, with *N*-isopropylacrylamide (NIPAAm) and acrylamide (AAm) as monomers, *N,N*-methylenebisacrylamide (BIS) as the crosslinker, and chitosan. The silane functionalization of magnetic nanoparticles (MNPs) was crucial for improving their compatibility, surface reactivity, and stability, facilitating better integration into the polymer matrix and preventing aggregation. SEM, TEM, and FTIR were used to verify the structure, morphology, and composition of the nanocomposite. The PAC-MNPs exhibited remarkable superparamagnetic properties and had an average size of approximately 150 nm. When loaded with doxorubicin (DOX), a widely used anticancer drug, the nanoparticles demonstrated dual-responsive characteristics for drug release, with the highest release at 40 °C (~78%) compared to 37 °C (~33%), and at pH 6 (~55%) compared to pH 7.4 (~28%) after 21 days. The incorporation of chitosan improved nanoparticle biodegradability and pH responsiveness, making them suitable for in vivo applications. The addition of R11 peptides, which specifically target prostate cancer cells, increased PAC-MNPs uptake by PC3 prostate cancer cells. Cytocompatibility studies showed no significant toxicity at concentrations up to 500 μg·mL^−1^ after 24 h of incubation with human dermal fibroblasts and normal prostate epithelial cells. Pharmacokinetic analysis demonstrated temperature-dependent cytotoxicity against prostate cancer cells, supporting the potential of PAC-MNPs for controlled drug delivery application.

Huang et al. developed a pH-responsive, polymer-coated hybrid Fe_3_O_4_ nanoparticle system for oral drug delivery, employing a layer-by-layer (LbL) assembly of casein (CN)-coated magnetic nanoparticles, reported as CN-DOX-IO, with the aim to enhance drug stability and bioavailability in the gastrointestinal tract [166]. This system incorporated Fe_3_O_4_ NPs carrying DOX and indocyanine green, initially coated with an amphiphilic copolymer of poly(maleic acid) and octadecene, and subsequently enclosed with an outer casein layer. In the acidic environment of the stomach (pH ~2), the casein coating provided stability, preventing premature degradation of the drug-loaded nanoparticles and enabling drug release in response to enzymatic activity at the neutral pH of the small intestine (pH ~7) in the presence of trypsin. The synthesis involved sequential steps: DOX loading onto Fe_3_O_4_ NPs, copolymer coating, and casein encapsulation via glutaraldehyde cross-linking. Characterization techniques, including gel electrophoresis, TEM, DLS, and UV–Vis spectroscopy confirmed nanoparticle size, stability, and DOX loading efficiency. Ex vivo and in vitro studies showed improved drug absorption and cellular uptake in the small intestine, while in vivo imaging in mice confirmed enzyme-responsive drug release. Casein was selected for its ability to form micelle-like structures, improving drug stability and bioavailability. DOX was chosen for its well-known chemotherapeutic efficacy and intrinsic fluorescence for tracking. The LbL-assembled casein-coated Fe_3_O_4_ NPs offer potential advantages for oral drug delivery, including reduced side effects, controlled drug release, and improved therapeutic efficacy, making them a promising candidate for future pharmaceutical applications.

A clearer picture emerges when these Fe_3_O_4_ drug delivery studies are viewed alongside the polymer-coated systems discussed for Au, Ag, Cu, and Pd nanoparticles. Whereas Au and Pd carriers often rely on optical activation or receptor-mediated uptake, and Ag and Cu platforms frequently emphasize antimicrobial or oxidative mechanisms, Fe_3_O_4_ nanoparticles provide the distinct advantage of magnetic guidance and localization. The PAC-coated Fe_3_O_4_ system demonstrates how thermo- and pH-responsive polymers can enable finely tuned, environment-dependent release in tumor settings, while the casein-based coated Fe_3_O_4_ nanoparticles illustrate how protective protein layers can enable oral administration and enzyme-triggered drug release in the gastrointestinal tract. In both cases, the polymer shell stabilizes the magnetic core, prevents aggregation, supports biological compatibility, and governs release kinetics under physiologically relevant conditions. These findings indicate that the effectiveness of Fe_3_O_4_ nanocarriers depends on designing polymer coatings that integrate magnetic control with stimulus sensitivity, allowing precise spatial and temporal delivery of therapeutic agents [165,166].

#### 4.1.2. Polymer-Coated Fe_3_O_4_ NPs in Magnetic Resonance Imaging (MRI)

Polymer-coated Fe_3_O_4_ NPs exhibit superior biocompatibility and distinct magnetic properties, making them effective MRI contrast agents. MRI contrast agents are substances injected into the body to enhance the visibility of specific tissues, blood vessels, or abnormalities during an MRI scan. These agents alter the relaxation rates of nearby water molecules, improving the contrast between different tissue types in the images. This enhancement facilitates the differentiation of abnormal tissues from normal tissues [190].

MRI utilizes two types of relaxation times, known as *T*_1_ and *T*_2_, to describe how protons in tissues return to equilibrium following disruption by a magnetic pulse. Transverse relaxation (*T*_2_) refers to the loss of coherence in the transverse plane, while longitudinal relaxation (*T*_1_) describes the time required for protons to realign with the external magnetic field. *T*_2_-weighted images are useful for detecting anomalies such as fluid accumulation, whereas *T*_1_-weighted images provide detailed anatomical information. Due to their superparamagnetic nature, Fe_3_O_4_ NPs may generate strong local magnetic fields, altering the relaxation times of adjacent water protons and improving the contrast of MRI images. Fe_3_O_4_ and γ-Fe_2_O_3_ NPs, characterized by high saturation magnetization, may also create strong magnetic field gradients that intensify the *T*_2_ relaxation effect, leading to darker images in *T*_2_-weighted MRI scans. This property is particularly useful for identifying anomalies such as tumors and vascular lesions [159,191,192,193]. For instance, PEG-coated Fe_3_O_4_ NPs can be administered intravenously for tumor imaging. The PEG coating extends the circulation time of the nanoparticles via the enhanced permeability and retention (EPR) effect, allowing them to accumulate in tumor tissue. Once localized within the tumor, the strong magnetic properties of Fe_3_O_4_ NPs enhance contrast in *T*_2_-weighted MRI images, improving tumor boundary visualization and facilitating early diagnosis and treatment planning. Additionally, the polymer coating acts as a protective layer, reducing potential toxicity and minimizing immune responses. Biocompatible polymers such as PEG are commonly used in medical applications due to their ability to evade the immune system and their non-toxic nature. The combination of enhanced imaging capabilities and a favorable safety profile makes polymer-coated Fe_3_O_4_ NPs promising MRI contrast agents.

**Figure 13:**
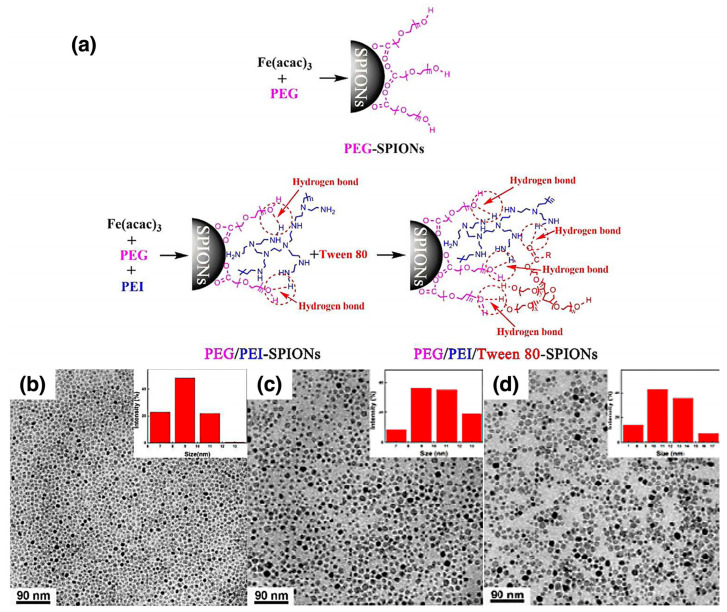
(**a**) Schematic representation of the synthesis and possible surface coating of SPIONs. (**b**–**d**) TEM images and corresponding size distributions of PEG-SPIONs, PEG/PEI-SPIONs, and PEG/PEI/Tween 80-SPIONs, respectively. Reproduced with permission from Ref. [167]. Copyright 2015 Elsevier.

Xie et al. investigated the synthesis and MRI contrast properties of superparamagnetic iron oxide nanoparticles (SPIONs) coated with different polymers, including PEG, PEG/PEI, and PEG/PEI/Tween 80 [167]. The synthetic scheme and potential interactions are illustrated in Figure 13a. Iron(III) acetylacetonate (Fe(acac)_3_) was thermally decomposed in PEG, with or without PEI, to produce PEG-SPIONs. In this process, PEG was directly grafted onto the SPION surface (i.e., in situ approach [68,194]), improving their colloidal stability. PEG was selected for its water solubility and its ability to enhance the aqueous solubility of hydrophobic drugs, thereby extending nanoparticle circulation time in vivo. PEI, a cationic polymer, was incorporated due to its ability to electrostatically interact with anionic molecules, forming compact nanoscale polyelectrolyte complexes that efficiently condense DNA and facilitate high-efficiency transfection in various cell lines. The primary interaction between PEG and PEI involves hydrogen bonding between the amine (-NH_2_) and hydroxyl (-OH) groups of PEI, with additional van der Waals forces between their polymer chains stabilizing the association on the SPION surface. To further improve water dispersibility and nanoparticle stability, Tween 80, a nonionic surfactant, was added as a coating agent. Tween 80 enhances the solubility of hydrophobic compounds and stabilizes emulsions, making it well-suited for biomedical applications. During the synthesis of PEG/PEI/Tween 80-SPIONs, hydrogen bonds formed between Tween 80, PEG, and PEI. The resulting SPIONs showed remarkable colloidal stability in deionized water, with mean hydrodynamic diameters of 19.5, 21.0, and 24.0 nm, and zeta potentials of −5.0, 35.0, and 19.0 mV for PEG-SPIONs, PEG/PEI-SPIONs, and PEG/PEI/Tween 80-SPIONs, respectively. TEM confirmed the formation of well-defined cubic spinel magnetite structures (Figure 13b–d). The average diameters of PEG-SPIONs, PEG/PEI-SPIONs, and PEG/PEI/Tween 80-SPIONs were 9.3 ± 1.6 nm, 10.3 ± 1.9 nm, and 11.5 ± 2.2 nm, respectively. FTIR verified the presence of PEG, PEI, and Tween 80 on the SPION surfaces. Superconducting quantum interference device (SQUID) measurements revealed that all SPION variants exhibited superparamagnetic behavior with saturation magnetizations of 53.3, 50.4, and 47.3 emu/g for PEG-SPIONs, PEG/PEI-SPIONs, and PEG/PEI/Tween 80-SPIONs, respectively. In vivo MRI studies on Kunming (KM) mouse brains demonstrated the potential of these SPIONs as MRI contrast agents. In comparison to PEG/PEI-SPIONs and PEG/PEI/Tween 80-SPIONs, PEG-SPIONs offered superior contrast enhancement and exhibited slower clearance by the mononuclear phagocytic system. The nanoparticles facilitated vascular imaging in different brain regions, with the PEG coating contributing to prolonged circulation time and reduced mononuclear phagocytic system uptake.

In summary, PEG-SPIONs, PEG/PEI-SPIONs, and PEG/PEI/Tween 80-SPIONs exhibit distinct surface functionalities and zeta potentials, influencing their MRI contrast properties. Due to their prolonged circulation time and low toxicity, these nanoparticles can be considered promising candidates for further research and development in biomedical imaging.

**Figure 14:**
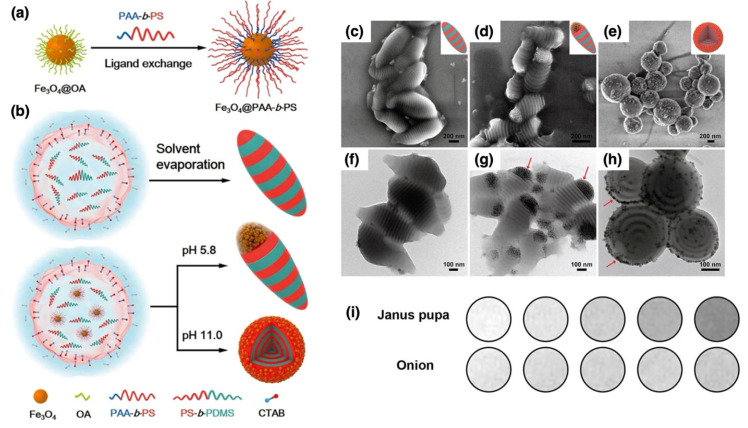
(**a**) Schematic illustrations of the ligand exchange process for preparing Fe_3_O_4_@PAA-*b*-PS NPs. (**b**) The emulsion–solvent evaporation method used to fabricate PS-*b*-PDMS microparticles with pH-responsive Fe_3_O_4_@PAA-*b*-PS NPs as cosurfactants. (**c**–**e**) SEM images of PS-*b*-PDMS/Fe_3_O_4_@PAA-*b*-PS microparticles at pH 5.8 and 11.0. (**f**–**h**) TEM images of PS-*b*-PDMS/Fe_3_O_4_@PAA-*b*-PS microparticles at pH 5.8 and 11.0. (**i**) *T*_2_-weighted MRI images of two typical PS-*b*-PDMS/Fe_3_O_4_@PAA-*b*-PS microparticles. Insets show BCP microparticle morphologies (PS in red, PDMS in turquoise). Reproduced with permission from Ref. [168]. Copyright 2020 American Chemical Society.

As another promising candidate for MRI contrast agents, Hou et al. developed a novel method for fabricating deformable block copolymer (BCP) microparticles with excellent shape control by manipulating the localization of pH-responsive nanoparticles [168]. The BCP employed in this study was polystyrene-*b*-poly(dimethylsiloxane) (PS-*b*-PDMS), synthesized using the ATRP method. To prepare Fe_3_O_4_ NPs, a thermal decomposition technique was used, specifically yielding oleic acid (OA)-capped Fe_3_O_4_ NPs (Fe_3_O_4_@OA). This process involved heating metal precursors in the presence of solvents and surfactants until decomposition into nanoparticles occurred. Subsequently, a ligand exchange technique following the “grafting to” method was used to modify the nanoparticle surface with poly(acrylic acid)-b-polystyrene (PAA-*b*-PS), as shown in Figure 14a. An emulsion–solvent evaporation technique was employed to incorporate the PS-*b*-PDMS BCP with the surface-modified iron oxide nanoparticles (Fe_3_O_4_@PAA-*b*-PS), as shown in Figure 14b. The localization of these pH-responsive nanoparticles was controlled under acidic (pH 5.8) and basic (pH 11.0) conditions. Under acidic conditions, the carboxylic acid groups in PAA were protonated, rendering the PAA segment hydrophobic. This led to nanoparticle aggregation within the PS domains, forming Janus pupa-like microparticles. Conversely, at high pH, the deprotonation of carboxylic acid groups rendered the PAA segment hydrophilic and negatively charged, prompting the nanoparticles to migrate to the oil/water interface and form onion-like microparticles. By simulating various biological conditions through pH variations, the potential in vivo behavior of these materials was assessed.

Shape control played a crucial role in this approach. The denser packing of Fe_3_O_4_ NPs in Janus pupa-like microparticles established a strong correlation between microparticle morphology and MRI contrast performance. This shape control enabled precise nanoparticle localization within the microparticles, either at the oil/water interface or within the PS domains, depending on pH conditions. SEM and TEM demonstrated that increasing the pH shifted Janus pupa-like structures toward spherical onion-like structures, as depicted in Figure 14c–h. Additional analyses, including DLS and zeta potential measurements, confirmed the localization of Fe_3_O_4_ NPs and their impact on microparticle shape. The *T*_2_-weighted MRI performance of the hybrid BCP microparticles, loaded with Fe_3_O_4_ NPs, was morphology-dependent. Janus pupa-like microparticles showed greater MRI contrast compared to the onion-like microparticles due to their denser Fe_3_O_4_ nanoparticle packing, as shown in Figure 14i. The morphology-dependent behavior suggests that these hybrid microparticles hold potential as contrast agents for MRI.

A comparison of these MRI-focused systems shows that polymer selection and structural arrangement play central roles in determining imaging performance. In the PEG-, PEG/PEI-, and PEG/PEI/Tween 80 coated SPIONs, differences in surface charge and hydration layer thickness influenced circulation time and contrast intensity, with PEG coatings providing the most favorable stability and persistence in vivo. In the block copolymer microparticle system, contrast efficiency was instead governed by the spatial distribution and packing density of the Fe_3_O_4_ nanoparticles, where the Janus pupa-like morphology produced stronger *T*_2_ signal effects than the onion-like configuration. Together, these results emphasize that effective MRI contrast design can be achieved either through careful tuning of surface chemistry at the nanoparticle interface or through controlled nanoparticle localization within larger polymer assemblies, depending on the intended imaging environment and performance requirements [167,168].

#### 4.1.3. Polymer-Coated Fe_3_O_4_ NPs in MRI-Guided Drug Delivery

The use of polymer-coated Fe_3_O_4_ NPs in MRI-guided drug delivery holds significant promise for cancer treatment. In a typical application, an external magnetic field can be used to direct chemotherapeutic agent-loaded nanoparticles toward a tumor. The strong magnetic characteristics of Fe_3_O_4_ NPs enhance MRI contrast after the nanoparticles accumulate in tumor tissue, allowing for precise confirmation of the drug delivery site. Additionally, these nanoparticles can be engineered to release the drug in response to the tumor microenvironment, ensuring both targeted and efficient treatment.

Yang et al. conducted a comprehensive study on the cooperative assembly of magneto-nanovesicles (MVs) designed to improve MRI-guided drug-delivery systems [169]. These nanovesicles were created by embedding SPIONs within polymeric membranes, with the primary objective of controlling membrane permeability and thickness. The MVs were designed to serve a dual function: transporting therapeutic drugs and acting as MRI contrast agents. Two amphiphilic block copolymers (BCPs), poly(styrene)-*b*-poly(acrylic acid) (PS-*b*-PAA) and thiol-ended poly(styrene)-*b*-poly(ethylene oxide) (PS-PEO-SH), were used to cooperatively build the MVs. RAFT polymerization was used to synthesize PS-PEO-SH, referred to as thiol-terminated BCPs, which were subsequently functionalized with maleimide-terminated dopamine to form dopamine-terminated BCPs through a Michael addition reaction. The oleic acid originally capping the nanoparticles was replaced by the dopamine group, which chelated iron atoms on the SPION surface, resulting in the “grafting to” method. The PEO block of PS-PEO-SH enhanced vesicle stability and prevented aggregation, while the hydrophobic PS block integrated into the membrane core. Following polymer modification, the SPIONs were mixed with PS-*b*-PAA, leading to their self-assembly into MVs. With the hydrophilic PAA block stabilizing the outer surface in the aqueous environment and the hydrophobic PS block forming the core, the PS-*b*-PAA copolymer provided structural stability to the vesicle membrane.

To optimize the drug release patterns of the MVs, it was necessary to regulate membrane permeability and thickness, which was achieved through this polymer system. The permeability and drug release properties of the MVs were directly influenced by the adjustable membrane thickness, which ranged from 9.8 nm to 93.2 nm, by adjusting the weight ratio of PS-*b*-PAA to SPIONs. This modulation resulted in a transition from monolayer MVs (MoMVs) to double-layered MVs (DoMVs), and multilayered MVs (MuMVs).

Comprehensive characterization was conducted to evaluate the MVs’ structural properties, magnetic functionality, and drug delivery potential. TEM confirmed the formation of spherical vesicles with embedded SPIONs and allowed precise measurement of membrane thickness. DLS revealed a narrow size distribution, indicating strong colloidal stability in aqueous environments. Magnetic measurements using a SQUID magnetometer validated the superparamagnetic behavior of the SPIONs, which is essential for enhancing MRI contrast. Zeta potential analysis further supported the stability of the MVs in suspension by demonstrating a stable surface charge conducive to dispersion in biological fluids. For drug delivery evaluation, the chemotherapeutic agent doxorubicin (DOX) was encapsulated within the MVs. The PS-*b*-PAA component allowed fine-tuning of the drug release profile by regulating membrane thickness: thinner membranes facilitated rapid drug release, while thicker membranes offered sustained release. To enhance tumor targeting, the MVs were conjugated with RGD peptides, which bind to integrin receptors overexpressed on tumor cells. In vivo studies showed that the RGD-functionalized-DOX-loaded MuMVs successfully targeted tumor sites, as evidenced by enhanced antitumor activity and MRI signals in a mouse model. The combination of magnetic targeting via SPIONs and active targeting via RGD peptides resulted in an efficient drug-delivery system capable of precisely localizing therapeutic agents, enabling real-time monitoring of drug delivery.

In summary, MVs with tunable structural features significantly improve their dual role as drug-delivery vehicles and MRI contrast agents.

#### 4.1.4. Polymer-Coated Fe_3_O_4_ NPs in Gene Delivery

Beyond drug delivery, polymer-coated Fe_3_O_4_ NPs also show significant potential for gene delivery applications. Gene delivery involves transporting genetic material, such as DNA or RNA, into cells to treat diseases, study gene function, or correct genetic flaws. The magnetic properties, biocompatibility, and functional versatility of polymer-coated Fe_3_O_4_ NPs make them excellent gene delivery vehicles.

Surface functionalization of these nanoparticles allows for efficient binding of genetic material. For instance, Fe_3_O_4_ NPs can be coated with cationic polymers, such as PEI, which carry a positive surface charge that facilitates electrostatic binding with negatively charged DNA or RNA molecules. This binding protects genetic material from degradation and enhances cellular uptake.

**Figure 15:**
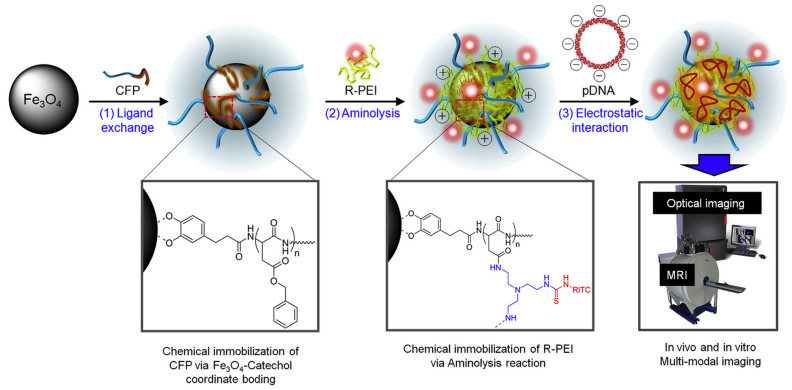
Design of multi-modal transfection agent (MTA) and its application in a multi-modal imaging system. Reproduced with permission from Ref. [170]. Copyright 2014 Elsevier.

Park et al. developed a multi-modal transfection agent (MTA) based on monodisperse magnetic nanoparticles to improve gene delivery efficiency and enable real-time tracking of human mesenchymal stem cells (hMSCs) [170]. Their study addressed critical challenges in stem cell therapy, including non-invasive monitoring of transplanted cells and controlled gene expression. The MTA was synthesized using a two-step functionalization process (Figure 15). Initially, Fe_3_O_4_ NPs were functionalized with catechol-functionalized polypeptide (CFP) using a ligand exchange approach, possibly following the “grafting to” method facilitated by the chemical immobilization of catechol groups on Fe_3_O_4_ NPs [51,52,195]. The catechol groups in CFP strongly bound to the Fe_3_O_4_ surface, replacing the original hydrophobic ligands and ensuring nanoparticle stability in biological environments. CFP also provided a functional substrate for further bioactive modifications. In the second step, rhodamine-labeled polyethyleneimine (R-PEI) was synthesized by covalently attaching rhodamine to PEI via amide bond formation. This modification conferred fluorescence to the MTA, enabling optical imaging of transfected cells. The R-PEI was then grafted onto the CFP-coated nanoparticles through aminolysis, forming stable covalent bonds between reactive sites on CFP and R-PEI. This functionalization imparted dual imaging capability to the MTA—fluorescence for optical imaging and superparamagnetic properties for MRI. Characterization confirmed successful nanoparticle synthesis and functionalization. TEM imaging revealed monodisperse, uniformly spherical nanoparticles with an average diameter of ~40 nm. DLS analysis corroborated these findings, showing a hydrodynamic size of ~40 nm. FTIR identified characteristic absorption peaks corresponding to rhodamine, polypeptide backbones, and catechol groups, confirming effective surface modification. SQUID magnetometry demonstrated strong saturation magnetization and superparamagnetic behavior, essential for MRI-based tracking of transfected cells. Zeta potential analysis revealed a positive surface charge, indicating the effective immobilization of positively charged PEI onto CFP-functionalized Fe_3_O_4_ NPs. In vitro studies showed that MTA/DNA complexes exhibited high transfection efficiency in hMSCs while maintaining lower cytotoxicity compared to conventional PEI-based transfection agents. Rhodamine fluorescence enabled optical imaging of transfected cells, confirming gene delivery. In vivo studies in mice transplanted with MTA-transfected hMSCs further demonstrated the dual imaging capability of the MTA. Using both MRI and optical imaging techniques, the transfected cells were successfully monitored in real time over a period of 14 days. MRI provided high-resolution anatomical features, ensuring precise tracking of the transplanted cells within the host tissue, while fluorescence imaging enabled continuous visualization of cell distribution and movement.

This strategy provides a flexible platform that can be extended to additional bioactive compounds, broadening its applications in regenerative medicine, gene therapy, and diagnostics. By integrating magnetic resonance and optical imaging into a single transfection agent, it enables precise monitoring and control of stem cell therapy, paving the way for more effective and personalized treatment approaches.

#### 4.1.5. Polymer-Coated Fe_3_O_4_ NPs in Hyperthermia

Polymer-coated Fe_3_O_4_ NPs have emerged as a highly effective tool in biomedical research, particularly for cancer therapy, as discussed in previous sections. The combination of drug delivery and heat therapy is a promising dual-therapy strategy that enhances therapeutic efficacy while minimizing damage to healthy tissues [196,197,198]. Inducing hyperthermia by raising the body temperature to a specific point (typically 41–44 °C) can selectively damage or destroy cancer cells without harming healthy cells [197]. Cancer cells are particularly vulnerable to heat, especially when combined with radiation or chemotherapy. Fe_3_O_4_ NPs can generate localized heat when exposed to an alternating magnetic field, making them well-suited for hyperthermia-based treatments [199,200].

**Figure 16:**
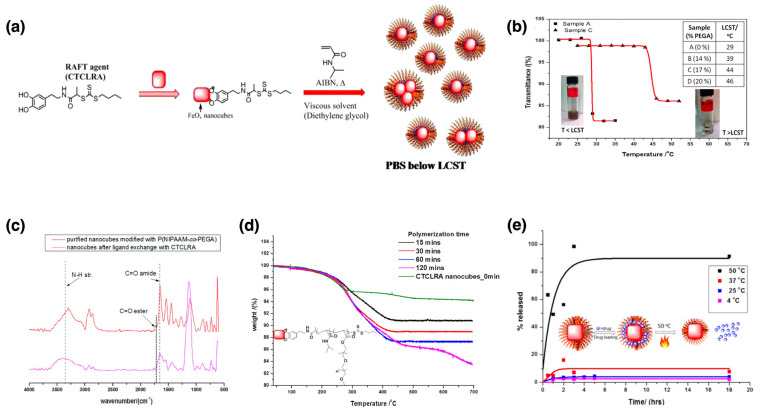
(**a**) Schematic representation of the synthetic approach used to create thermoresponsive cubic-IONPs in viscous solvent (diethylene glycol) and their dispersion in an aqueous solution. (**b**) Turbidimetric analysis of PNIPAAM-coated cubic-IONPs (Sample A) and PNIPAAM-co-PEGA functionalized cubic-IONPs (Sample C). (**c**) FTIR spectra of cubic-IONPs functionalized with CTCLRA before and after polymerization with PNIPAAM/PEGA in diethylene glycol. (**d**) TGA of PNIPAAM-co-PEGA functionalized cubic-IONPs (structure shown in the inset) at different polymerization times. (**e**) Drug release profile of thermoresponsive PNIPAAM-co-PEGA coated cubic-IONPs in a water bath at various temperatures. Reproduced with permission from Ref. [171]. Copyright 2015 American Chemical Society.

Kakwere et al. investigated the functionalization of iron oxide nanocubes (cubic-IONPs) with thermoresponsive and pH-responsive polymer coatings to develop advanced nanohybrids for heat-triggered drug delivery and hyperthermia [171]. Cubic-IONPs were chosen due to their exceptionally high specific absorption rate, making them highly effective for hyperthermia applications. However, strong interparticle interactions among these cubic-IONPs posed challenges for functionalization. To overcome this, RAFT polymerization was employed, enabling controlled polymer chain growth directly from the nanoparticle surface and resulting in homogeneous, high-density polymer coatings. As illustrated in Figure 16a, cubic-IONPs were initially functionalized with a catechol-bearing RAFT agent (CTCLRA), which strongly bound to the iron oxide surface. This modification converted the nanoparticle surface into an active site for controlled monomer polymerization using the “grafting from” method with AIBN as the initiator. Functionalized nanoparticles then served as initiation sites for polymerizing monomers such as vinylpyridine (VP) and *N*-isopropylacrylamide (NIPAAM) directly from the nanoparticle surface. The goal of this study was to graft poly(*N*-isopropylacrylamide) (PNIPAAM) onto cubic-IONPs to create thermoresponsive nanohybrids. To ensure their stability at physiological temperatures and enable hyperthermia-triggered drug release, PNIPAAM was copolymerized with polyethylene glycol methyl ether acrylate (PEGA), raising the LCST of the nanohybrids above 37 °C. In addition to thermoresponsive coatings, PVP was polymerized onto cubic-IONPs to create pH-responsive nanohybrids. Due to the protonation of pyridine groups, these nanohybrids exhibited water solubility below pH 5 but precipitated above pH 5, providing an additional mechanism for controlled drug release.

Extensive characterization was performed to confirm successful polymerization and assess the potential of functionalized cubic-IONPs for biomedical applications. TEM and cryo-TEM analyses confirmed that the cubic morphology of IONPs was preserved even after polymer coating, while DLS measurements indicated an increase in hydrodynamic diameter, confirming successful polymer functionalization. Turbidimetric analysis, conducted by monitoring changes in optical transmittance with increasing temperature, validated the LCST of the thermoresponsive polymer-functionalized nanoparticles, which is essential for temperature-sensitive drug release applications. The LCST of PNIPAAM-coated cubic-IONPs was approximately 29 °C, at which the polymer became insoluble, causing nanoparticle aggregation and precipitation (Figure 16b). In contrast, PNIPAAM-co-PEGA functionalized cubic-IONPs exhibited a tunable LCST that could be increased above 37 °C by adjusting the NIPAAM-to-PEGA ratio. This ensured nanoparticle stability under physiological conditions and suitability for hyperthermia-triggered drug release. In Figure 16c, the FTIR spectra exhibited distinctive absorption peaks, including amide (C=O and N–H) and ester (C=O) peaks, suggesting the presence of a thermoresponsive polymer shell on the nanocube’s surface. The TGA data, shown in Figure 16d, revealed a noticeable weight loss consistent with the degradation of the polymer layer, which typically began at 300 °C. SQUID magnetometer magnetization experiments confirmed that the polymer-coated cubic-IONPs retained their superparamagnetic properties, which are essential for hyperthermia applications. Cytotoxicity tests were conducted to assess the biocompatibility of the polymer-coated nanocubes. Furthermore, for drug release experiments, the polymer-coated nanohybrids were loaded with DOX as the model drug. As shown in Figure 16e, these nanohybrids demonstrated non-toxicity in experiments on Kb cells, a subline of HeLa cells, with cell viability remaining above 90% at tested nanoparticle doses.

Overall, these dual thermoresponsive and pH-responsive nanohybrids, synthesized via RAFT polymerization directly from the cubic-IONP surface, hold great promise for cancer treatment, particularly in hyperthermia and chemotherapy with stimuli-triggered drug release. This functionalization approach effectively addresses the challenges associated with highly interacting cubic-IONPs and offers a pathway for developing intelligent, stimuli-responsive nanomaterials for biomedical applications.

### 4.2. Titanium Dioxide Nanoparticles (TiO_2_ NPs)

Titanium dioxide nanoparticles (TiO_2_ NPs) have gained significant attention in recent years due to their distinctive physicochemical properties, enabling a wide range of applications. The production and modification of these nanoparticles have facilitated advancements across various scientific and industrial domains, particularly in biological applications [201,202].

TiO_2_ exists in three primary crystalline forms: anatase, rutile, and brookite, each possessing unique physical and chemical properties. Anatase, characterized by a tetragonal crystal structure and a bandgap energy of ~3.2 eV, is the most photoactive polymorph. Rutile, a more thermodynamically stable tetragonal structure, has a slightly lower bandgap of ~3.0 eV. Brookite, the least common polymorph, has an orthorhombic structure with properties intermediate between anatase and rutile [203,204].

The functionality of TiO_2_ NPs is significantly influenced by their surface chemistry. The presence of hydroxyl (-OH) groups on the surface renders TiO_2_ hydrophilic, permitting further chemical modifications [204]. These surface hydroxyl groups also take part in photocatalytic reactions, contributing to the production of ROS under light irradiation. The electrical structure of TiO_2_ NPs is responsible for their remarkable photocatalytic activity. When exposed to light with an energy equal to or greater than its bandgap, electrons are excited from the valence band to the conduction band, leaving behind positively charged holes. These electron-hole pairs migrate to the surface, where they participate in redox reactions. The excited electrons reduce oxygen molecules to superoxide radicals (O_2_^−^•), while the holes oxidize water or hydroxyl ions to hydroxyl radicals (•OH) [205,206]. The high reactivity of these ROS enables TiO_2_ NPs to degrade organic contaminants and exhibit strong antibacterial properties.

The photocatalytic activity of TiO_2_ NPs can be further enhanced through doping, especially when exposed to visible light. Doping is the process of introducing foreign elements, such as sulfur, nitrogen, or transition metals, into the TiO_2_ lattice. These dopants reduce the bandgap energy and introduce mid-gap states, thereby extending the absorption spectrum of TiO_2_ into the visible light range [207,208,209].

#### 4.2.1. Polymer-Coated TiO_2_ NPs in Bone Tissue Regeneration

Polymer-coated TiO_2_ NPs can also be functionalized with bioactive substances such as peptides and growth factors to enhance their osteogenic potential. These functionalized nanoparticles can promote the body’s natural bone-healing process, aiding in the treatment of bone abnormalities and fractures. TiO_2_ NPs can be incorporated into orthopedic implant coatings, providing antimicrobial properties that lower infection risks and improve implant integration with surrounding bone tissue.

Rezk et al. investigated the combination of simvastatin (SIM)-loaded poly(ε-caprolactone) (PCL) and polyaniline (PANI)-coated TiO_2_ NPs (TiO_2_/PANI) using electrospinning to create a composite nanofiber scaffold for bone tissue regeneration [172]. TiO_2_ NPs were coated with PANI via oxidative chemical polymerization. In this study, the formation of hybrid TiO_2_/PANI nanomaterials is likely driven by the physical adsorption of PANI polymers onto TiO_2_ NPs, possibly facilitated by hydrogen bonding between them [47,48,49,210]. PCL was chosen for its biocompatibility and gradual biodegradability, while SIM was included for its ability to promote bone growth and reduce inflammation. Also, PANI polymers are very well-known materials due to their electrical conductivity and stability [211]. Nanofiber composites offer several advantages, including a high surface area-to-volume ratio, which mimics the extracellular matrix (ECM) found in natural tissues and improves cell adhesion, proliferation, and differentiation. In addition, the fibrous structure provides interconnected porosity, facilitating waste elimination and the exchange of oxygenated blood, which is vital for maintaining cell viability and function. Nanofibers can also encapsulate drugs, allowing for controlled and prolonged release, thereby improving therapeutic efficacy. The mechanical properties of fibers can be tailored to provide the necessary strength and flexibility required for certain uses, including bone tissue regeneration. Electrospinning was key technique in this study for generating continuous nanofibers with controlled morphology. This method enabled the production of composite nanofibers with enhanced mechanical properties and regulated drug release profiles. The composite’s crystalline structure and the presence of PANI were confirmed through FTIR and XRD analyses, while field-emission SEM (FE-SEM) images revealed bead-free fibers with a uniform nanoparticle distribution. TGA showed improved thermal stability of the composite nanofibers. According to in vitro drug release studies, an increased concentration of TiO_2_/PANI nanoparticles facilitated a faster release of SIM. Biomimetic mineralization studies conducted in simulated bodily fluid (SBF) indicated the formation of a hydroxyapatite coating on the nanofiber surface, suggesting their potential for bone regeneration. Cell culture experiments with mouse osteoblasts (MC3T3-E1) demonstrated excellent cell viability, proliferation, and adhesion, confirming good biocompatibility. Based on these findings, SIM-loaded TiO_2_/PANI-PCL composite nanofibers offer a controlled drug release mechanism along with superior mechanical and bioactive properties, making them a promising scaffold for bone tissue regeneration applications.

Overall, this study shows that polymer-coated TiO_2_ nanoparticles can strengthen scaffold structure while also promoting osteogenic activity and controlled drug release. The combination of PCL, PANI, and simvastatin supported cell adhesion and mineralization, indicating that such hybrid nanofiber systems are well-suited for bone tissue regeneration.

#### 4.2.2. Polymer-Coated TiO_2_ NPs in Biomedical Devices

TiO_2_ NPs have recently shown significant potential in biomedical applications, particularly in enzyme-catalyzed biofuel cells. In these biofuel cells, enzymes act as catalysts, converting the chemical energy stored in biofuels into electrical energy [212]. The large surface area, stability, and biocompatibility of TiO_2_ NPs make them ideal support materials for enzyme immobilization. Surface modification of TiO_2_ NPs can further enhance enzyme loading and activity, thereby improving biofuel cell efficiency [213].

To improve electron transport in biofuel cell (BFC) anodes, Haque et al. developed a polythiophene-titanium oxide (PTH-TiO_2_) nanocomposite [173]. BFC anodes play a crucial role in biofuel cells, as they facilitate the oxidation of biological fuels such as glucose. In enzyme-catalyzed biofuel cells, immobilized enzymes, such as glucose oxidase, oxidize glucose at the anode. During the conversion of glucose to gluconolactone, the enzyme generates protons and electrons. These electrons are transferred to the anode, where their efficient transport is critical for effective biofuel cell operation. Conductive materials such as TiO_2_ NPs and PTH can enhance this process. The electrons then flow through an external circuit, generating an electric current. Meanwhile, protons migrate through the electrolyte toward the cathode, where they combine with oxygen and electrons from the external circuit to form water, completing the process. TiO_2_ NPs were selected for this study due to their large surface area, superior photocatalytic properties, chemical stability, biocompatibility, and ability to facilitate electron transport. Their high surface area provides abundant active sites for enzyme immobilization, while their conductive and stable nature ensures efficient and long-lasting biofuel cell performance. PTH was selected as a polymer matrix for its chemical stability, processability, conductivity, and flexibility. Its conjugated structure improves its interactions with biological molecules, including enzymes and redox mediators, as well as TiO_2_ NPs, thereby enabling efficient electron transfer and stable immobilization.

**Figure 17:**
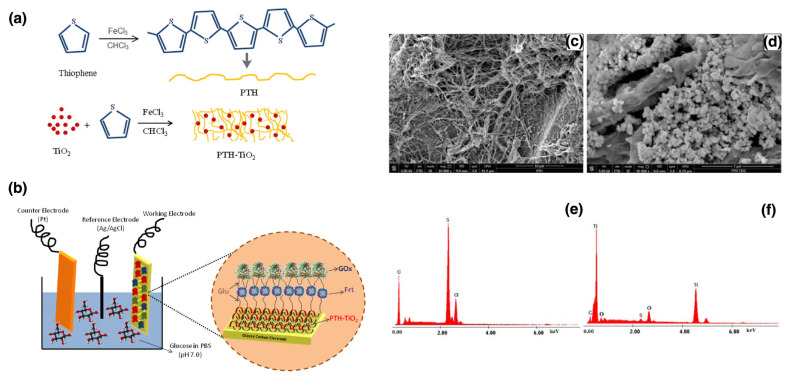
(**a**) Synthesis of PTH and PTH-TiO_2_ composite. (**b**) Schematic illustration of the PTH-TiO_2_/Frt/GOx bioanode. (**c**,**d**) SEM images of PTH and PTH-TiO_2_, respectively. (**e**,**f**) EDX spectra of PTH and PTH-TiO_2_, correspondingly. Reproduced with permission from Ref. [173]. Copyright 2015 Elsevier.

As shown in Figure 17a, the synthesis of the PTH-TiO_2_ nanocomposite involved the chemical oxidative polymerization of thiophene onto TiO_2_ NPs using ferric chloride (FeCl_3_) as the oxidant. In this process, thiophene monomers were first adsorbed onto the surface of TiO_2_ NPs, followed by the addition of FeCl_3_-saturated CHCl_3_ solution. This oxidized the monomers on the surface, initiating the propagation reaction to form the final polymer, possibly through “grafting from” method [56,57,58,59,214]. Figure 17b illustrates that the resulting nanocomposite effectively functioned as a stable bioanode by immobilizing glucose oxidase (GOx) and the redox mediator ferritin (Frt) on its conductive surface. SEM and EDX analyses (Figure 17c–f) confirmed the successful integration of TiO_2_ into the PTH matrix. SEM images revealed that PTH exhibited a globular structure, while PTH-TiO_2_ displayed a noodle-like fibrous morphology, with an average particle size of 70–90 nm. EDX analysis verified the presence of titanium in the composite, confirming its elemental composition. FTIR spectra confirmed the incorporation of PTH and TiO_2_, with characteristic peaks corresponding to Ti–O–Ti and PTH. XRD patterns showed the implementation of amorphous PTH crystalline to the crystalline PTH-TiO_2_ composite with the indication of pure PTH peak absence. Electrochemical analysis further validated the enhanced performance of the nanocomposite. Cyclic voltammetry (CV) data demonstrated a significant increase in current density, indicating efficient electron transfer and higher electrochemical activity due to the addition of TiO_2_ NPs. Stability tests demonstrated that the bioanode retained 88% of its initial current density after 14 days of storage at 4 °C, highlighting its durability.

In summary, the PTH-TiO_2_ nanocomposite serves as an effective conductive support material for improving the stability and performance of glucose-based biofuel cells. The PTH-TiO_2_/Frt/GOx bioanode exhibited enhanced stability, efficient electron transfer, and superior electrochemical performance, providing a promising strategy for developing robust and high-performance bioanodes.

### 4.3. Zinc Oxide Nanoparticles (ZnO NPs)

Zinc oxide nanoparticles (ZnO NPs) have garnered significant interest due to their diverse and adaptable properties, which stem from their unique chemistry [215]. ZnO is a wide bandgap semiconductor (~3.37 eV) that crystallizes in the wurtzite structure, a hexagonal lattice that imparts anisotropic characteristics to the material [216]. ZnO NPs exhibit remarkable optical properties, including strong UV absorbance and high photoluminescence efficiency, attributed to their structure and high exciton binding energy (60 meV) [215,217].

The surface chemistry of ZnO NPs plays a crucial role in their reactivity and functionality. Hydroxyl groups and other oxygen vacancies on the ZnO surface significantly influence their interactions with various materials, including contaminants, solvents, and biomolecules [218,219]. Due to their high surface-to-volume ratio, ZnO NPs exhibit enhanced surface reactivity, stability, and functionality across different applications [220,221]. Their surface properties can be further modified through functionalization, where specific molecules are bonded to the nanoparticle surface to improve stability, biocompatibility, or specificity for targeted applications. For instance, surface modification with organic ligands or polymers enhances ZnO NPs’ dispersion in biological and aquatic environments, reducing aggregation and increasing their efficacy in antibacterial treatments, environmental remediation, and the automotive industry, as discussed below.

#### 4.3.1. Polymer-Coated ZnO NPs in Antibacterial Treatments and Environmental Remediation

ZnO NPs are well known for their redox activity, particularly in photocatalytic processes. When exposed to UV radiation, ZnO NPs generate electron-hole pairs (e^−^/h^+^) as electrons are excited from the valence band to the conduction band. These charge carriers interact with adsorbed molecules on the ZnO surface, leading to the production of ROS, such as superoxide anions (O_2_^−^•) and hydroxyl radicals (•OH) [222]. The high reactivity of these ROS enables ZnO NPs to break down organic pollutants, making them effective photocatalysts for environmental remediation [223].

Polymer coatings on ZnO NPs can enhance their stability, dispersion, and controlled release of active agents, making them suitable for long-term antibacterial applications and environmental remediation. Bharathi et al. synthesized a chitosan-coated ZnO (CS-ZnO) nanocomposite using green chemistry with the bioflavonoid rutin as a reducing and stabilizing agent [174]. Chitosan, a biopolymer with antimicrobial, biodegradable, and biocompatible properties, was used to improve the antibacterial and photocatalytic efficiency of ZnO NPs. In this study, zinc sulfate was reduced to ZnO NPs using rutin, allowing for precipitation and purification. The resulting ZnO NPs were then incorporated into a chitosan solution to form the CS-ZnO nanocomposite. The interactions between chitosan and ZnO in the composite involved the hydrogen bonding between the hydroxyl and amine groups of chitosan and the surface of ZnO, leading to physisorption [47,48,49,224]. The formation of the CS-ZnO nanocomposite was confirmed using EDS, XRD, FE-SEM, DLS, FTIR, UV–Vis spectroscopy, and zeta potential analysis. The antibacterial activity was assessed using the disk diffusion method against both Gram-positive and Gram-negative bacterial pathogens. Results demonstrated strong antibacterial activity, particularly against Gram-negative bacteria, with *E. coli* showing the highest inhibition. This enhanced antibacterial effect was attributed to the interaction between the positively charged CS-ZnO nanocomposite and negatively charged bacterial membranes. In addition to its antimicrobial properties, the CS-ZnO nanocomposite exhibited significant photocatalytic activity in the degradation of Congo red and methylene blue dyes under sunlight. This performance was linked to the active surface area and small particle size, which improved the production, consumption, and transfer of photogenerated charge carriers. According to the study, the green synthesis approach used to prepare the chitosan-coated ZnO nanocomposite presents promising potential for environmental remediation and antibacterial treatments, particularly for the degradation of organic pollutants under sunlight. This work also highlights the potential of bioflavonoids, such as rutin, in synthesizing biopolymer-coated nanoparticles with enhanced functional properties.

The findings from this study demonstrate that chitosan plays a critical role in enhancing the functional performance of ZnO nanoparticles. By improving particle dispersion and promoting stronger interactions with bacterial membranes, the coating leads to more effective antibacterial activity. At the same time, the composite structure supports efficient charge transfer during photocatalysis, resulting in improved degradation of organic dyes. Together, these results highlight the value of polymer-coated ZnO nanoparticles as dual-purpose materials for antimicrobial applications and environmental pollutant removal.

#### 4.3.2. Polymer-Coated ZnO NPs in the Automotive Industry

ZnO NPs are being explored as fuel additives to improve engine efficiency. When used in lubrication systems, ZnO NPs form a protective tribofilm resistant to high temperatures and pressures, thereby reducing friction and wear between contact surfaces. Their nanoscale size facilitates better dispersion in lubricants, improving the smooth interaction of moving parts. ZnO NPs also exhibit outstanding antioxidant stability, preventing lubricant degradation at high temperatures. These properties make ZnO NPs valuable additives for enhancing engine performance and longevity, particularly in high-demand industrial applications [225,226].

To enhance the dispersion and stability of ZnO NPs in lubricants, polymer coatings have been investigated as a means to improve tribological behavior and overall performance. Vyavhare et al. developed and evaluated borate- and methacrylate-coated ZnO NPs (ZnOBM) as potential eco-friendly replacements for zinc dialkyl dithiophosphate (ZDDP), a hazardous anti-wear additive commonly used in automotive lubricants [175]. In this study, plasma polymerization was employed to synthesize ZnOBM, which were designed to form stable protective tribofilms, reducing friction and wear on ferrous surfaces. The performance of ZnOBM was tested under boundary lubrication conditions, either alone or in combination with lower concentrations of ZDDP. Tribological tests demonstrated that ZnOBM nanoadditives formed stable tribofilms, reducing friction and wear. Notably, ZnOBM improved wear resistance by up to 95% compared to base oil. Advanced surface characterization techniques such as AFM, X-ray absorption near-edge spectroscopy (XANES), and XPS revealed that ZnOBM formed heterogeneous tribofilms composed of zinc oxide, boron oxide, and iron borate, which improved tribological performance. The study also discovered synergistic interactions between ZnOBM nanoparticles and ZDDP, resulting in hierarchical tribofilms with superior wear protection. As a result, ZnOBM nanoparticles can significantly reduce harmful emissions while maintaining their anti-friction and anti-wear properties. This makes them a promising and environmentally friendly alternative to engine oils with high concentrations of ZDDP, contributing to the development of high-performance, eco-friendly lubricant additives for automotive applications.

This study demonstrates that polymer-coated ZnO nanoparticles form stable protective tribofilms that effectively reduce friction and wear, offering a cleaner and more efficient alternative to conventional lubricant additives in automotive systems.

### 4.4. Aluminum Oxide Nanoparticles (Al_2_O_3_ NPs)

Aluminum oxide nanoparticles (Al_2_O_3_ NPs), also known as alumina nanoparticles, have been extensively studied due to their exceptional chemical and physical characteristics [227]. Aluminum oxide exists in multiple polymorphic forms, with α-Al_2_O_3_ (corundum) and γ-Al_2_O_3_ being the most significant. The α-phase is highly valued for its chemical resistance, high hardness, and thermal stability, making it suitable for industrial and technological applications, including laboratory ware, furnace linings, and refractory materials [228,229]. The γ-phase, in contrast, is distinguished by its high porosity, large surface area, and catalytic activity, making it an essential material in catalysis and adsorption applications [230].

The surface chemistry of Al_2_O_3_ NPs is predominantly governed by hydroxyl groups, facilitating functionalization with various chemical groups or molecules [231]. Surface modification with polymers represents a significant advancement in expanding the applications of these nanoparticles. Polymer-coated Al_2_O_3_ NPs exhibit enhanced dispersibility and compatibility with different systems by combining the flexibility of the polymer matrix with the intrinsic properties of the alumina core. In addition, the polymer layer prevents undesirable surface reactions and aggregation, increasing the versatility of these nanocomposites for various applications. Recent advancements in polymer-coated Al_2_O_3_ NPs have demonstrated their potential in diverse fields, including cancer treatment, antibacterial applications in the food industry, and catalysis, as discussed in the following sections.

#### 4.4.1. Polymer-Coated Al_2_O_3_ NPs in Cancer Treatment

Polymer-coated Al_2_O_3_ NPs are valuable for both therapeutic and diagnostic applications due to their ability to induce cytotoxicity and facilitate selective protein adsorption. Their surface chemistry can be engineered to enhance biocompatibility and enable targeted drug delivery. ROS produced by γ-Al_2_O_3_ NPs can induce oxidative stress and cell death, particularly in cancer cells, which have elevated metabolic activity and reduced antioxidant defenses.

Polymer-coated Al_2_O_3_ NPs also play a critical role in selective protein adsorption, making them ideal for therapeutic and biosensing applications. The polymer coating can be engineered to bind specific proteins while preventing nonspecific adsorption, ensuring high specificity in protein interactions. This principle was demonstrated in a study by Rajan et al., where poly(γ-glutamic acid) (γ-PGA)-functionalized alumina nanoparticles (γ-PAN) were synthesized and characterized to assess their protein adsorption efficiency and cytotoxicity against PC-3 human prostate cancer cells [176]. In this study, Al_2_O_3_ NPs were synthesized using the precipitation-digestion method, where aluminum hydroxide was precipitated from aluminum sulfate using ammonia solution, followed by digestion to form alumina nanoparticles (AN). These nanoparticles were then functionalized with γ-PGA to enhance their surface properties and colloidal stability. Possible physisorption interactions, arising from hydrogen bonding between the carboxylic acid groups of γ-PGA and Al_2_O_3_ NPs, may contribute to the formation of this hybrid nanocomposite [47,48,49,232]. γ-PGA was chosen for its biocompatibility and biodegradability. By providing a hydrophilic and negatively charged surface, it improved the colloidal stability of nanoparticles, prevented aggregation, and enhanced their dispersion in biological environments. Additionally, the presence of numerous carboxylic acid groups in γ-PGA introduced a negative surface charge, allowing for the selective adsorption of positively charged proteins, such as lysozyme (LSZ).

Successful functionalization of the nanoparticles was confirmed by extensive characterization. XRD verified the spinel structure of γ-Al_2_O_3_, while TEM images revealed that the γ-PGA coating efficiently prevented agglomeration. Particle size analysis by TEM demonstrated that γ-PAN nanoparticles had a slightly larger mean particle size (6.7 nm) than uncoated AN (5.4 nm), confirming the presence of the polymer coating. Additionally, EDX detected an increase in carbon and oxygen content, further validating the successful attachment of γ-PGA to the nanoparticle surface. Zeta potential measurements showed that γ-PAN nanoparticles carried a negative charge across a wide pH range due to the ionized carboxylic acid groups of γ-PGA, whereas uncoated AN exhibited a positive charge. This surface charge variation influenced protein adsorption behavior, with uncoated AN preferentially adsorbing negatively charged BSA, while γ-PAN exhibited a higher affinity for positively charged LSZ. Cytotoxicity tests demonstrated that γ-PAN was more toxic to PC-3 prostate cancer cells than uncoated AN. The increased cytotoxicity was likely due to the enhanced colloidal stability and ROS generation of γ-PAN, which contributed to greater oxidative stress and cancer cell death.

Overall, the study highlights the potential of γ-PGA-functionalized AN as promising candidates for targeted cancer therapy and selective protein adsorption. These findings lay the foundation for further exploration in biomedical applications, particularly in nanoparticle-based cancer treatment.

#### 4.4.2. Polymer-Coated Al_2_O_3_ NPs in Antibacterial Applications in the Food Industry

Polymer-coated Al_2_O_3_ NPs hold great potential as antibacterial agents in the food industry, where preventing bacterial contamination is essential for ensuring food safety. The polymer coating enhances the antimicrobial properties of Al_2_O_3_ NPs by promoting ROS generation, which plays a key role in bacterial inactivation. Additionally, the polymer layer can be engineered to control the release of antimicrobial agents, extending the duration of antibacterial activity. This controlled release mechanism is particularly useful in food packaging applications, where sustained bacterial inhibition can help prolong the shelf life of perishable goods. Overall, the integration of polymer-coated Al_2_O_3_ NPs into food industry applications presents an innovative strategy for reducing bacterial contamination and improving food safety through advanced antimicrobial technologies.

In this context, Burmistrov et al. developed a composite coating composed of Al_2_O_3_ NPs and polytetrafluoroethylene (PTFE) for food industry applications where bacterial contamination is a concern. The objective was to create a bacteriostatic surface with low cytotoxicity, making it suitable for food-related environments [177]. The synthesis of Al_2_O_3_ NPs was carried out using a laser ablation technique, in which a high-purity aluminum plate was exposed to a pulsed ytterbium fiber laser in deionized water, producing Al_2_O_3_ NPs. The composite coating was then made by incorporating these nanoparticles into a PTFE varnish. PTFE was chosen as the polymer matrix due to its exceptional mechanical properties, thermal stability resulting from C–F bonding, hydrophobicity, and biocompatibility, making it ideal for applications in the food industry. Zeta potential measurements indicated a stable colloidal solution, with a zeta potential of ~50 mV, confirming the stability of the nanoparticle dispersion. AFM analysis verified that the composite coating was uniformly smooth and defect-free. The distribution of Al_2_O_3_ NPs within the PTFE matrix was further examined using modulation-interference microscopy (MIM), which demonstrated that the nanoparticles were evenly dispersed, forming clusters of varying sizes depending on nanoparticle concentration. At higher Al_2_O_3_ NP concentrations, the composite coating showed the ability to generate ROS, including hydrogen peroxide and hydroxyl radicals, which contributed to its antibacterial properties. The bacteriostatic effect of the coating was particularly effective against *E. coli*, a Gram-negative bacterium, inhibiting its growth by ROS-mediated oxidative stress.

Despite its strong antibacterial activity, the composite coating demonstrated low cytotoxicity in mammalian cells. In vitro experiments using mouse fibroblast cultures revealed that even at high nanoparticle concentrations, the coating did not significantly affect cell viability or proliferation. This balance between antibacterial efficacy and biocompatibility suggests that PTFE/Al_2_O_3_-NP composite coatings could be safely implemented in food industry applications and potentially extended to biomedical devices.

The study highlights that the physical entrapment and adsorption of Al_2_O_3_-NPs within the PTFE matrix contribute to the stability and functionality of the coating, ensuring long-term effectiveness in real-world applications.

#### 4.4.3. Polymer-Coated Al_2_O_3_ NPs in Catalysis

Polymer-coated Al_2_O_3_ NPs are highly effective catalysts for organic reactions due to the synergy between the catalytic properties of the alumina core and the functional versatility of the polymer coating. The polymer enhances catalytic performance by stabilizing catalytic species, improving the dispersion of active sites, and preventing deactivation, ultimately increasing overall catalytic activity. The ability to fine-tune catalytic properties through polymer modification allows for precise control over reaction conditions, making polymer-coated Al_2_O_3_ NPs well-suited for a wide range of organic transformations.

**Figure 18:**
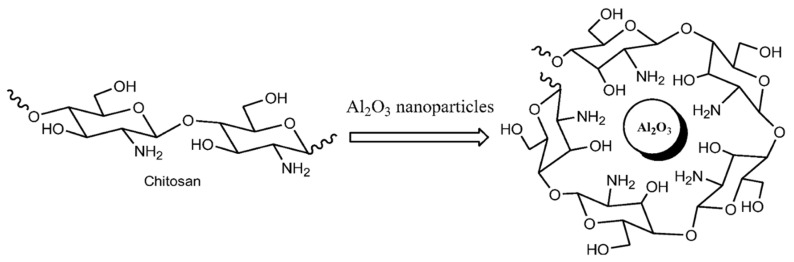
Schematic illustration of the chitosan-Al_2_O_3_ nanocomposite. Reproduced with permission from Ref. [178]. Copyright 2021 by the authors. Published by MDPI under the Creative Commons CC BY license.

Recently, Abdel-Naby et al. synthesized and characterized a chitosan-Al_2_O_3_ nanocomposite, as shown in Figure 18, to develop a green, heterogeneous catalyst for the synthesis of annulated imidazopyrazolthione derivatives [178]. Chitosan was chosen as the polymer matrix due to its non-toxicity, biodegradability, and intrinsic base catalytic properties. The incorporation of Al_2_O_3_ NPs into the chitosan matrix improved its catalytic efficiency while preventing issues such as gel formation during reactions. The formation of chitosan-coated Al_2_O_3_ NPs may be driven by possible physical adsorption through hydrogen bonding between the amino and hydroxyl groups of chitosan and Al_2_O_3_ NPs [44,45,46,218]. The structural and morphological characteristics of chitosan-Al_2_O_3_ nanocomposite were confirmed using FTIR, EDX, XRD, and emission scanning electron microscopy (ESEM), a specialized type of SEM. FTIR provided evidence of interactions between Al_2_O_3_ and chitosan, as indicated by changes in the distinctive chitosan peaks. XRD analysis confirmed the crystalline structure of the nanocomposite, while ESEM and EDX verified the successful incorporation of Al_2_O_3_ into the chitosan matrix, providing insights into its morphology and elemental composition, respectively.

Owing to its enhanced base catalytic activity, the chitosan-Al_2_O_3_ nanocomposite was used in the synthesis of imidazopyrazolylthione derivatives. Compared to conventional homogeneous catalysts, the heterogeneous nature of the composite resulted in higher catalytic efficiency, increased product yields, shorter reaction times, and simplified product separation. Furthermore, the nanocomposite demonstrated recyclability, as it could be recovered and reused up to four times without significant loss of catalytic activity. The antibacterial properties of the synthesized imidazopyrazolylthione derivatives were also evaluated. The results demonstrated broad-spectrum antibacterial activity against both Gram-positive and Gram-negative microorganisms. Molecular docking studies further confirmed strong binding affinities between the synthesized derivatives and bacterial protein targets, providing additional evidence for their potential as antibacterial drug candidates.

The study highlights the chitosan-Al_2_O_3_ nanocomposite as a versatile and environmentally benign catalyst with significant potential in green chemistry applications, particularly in the synthesis of biologically active substances. Its reusability, efficiency, and ability to facilitate eco-friendly reactions make it a promising candidate for sustainable catalytic processes.

To consolidate the information presented across various metal oxide systems, Table 4 summarizes representative polymer-coated Fe_3_O_4_, TiO_2_, ZnO, and Al_2_O_3_ nanoparticles, outlining their core compositions, polymer types, coating techniques, and functional outcomes. This overview bridges distinct oxide classes by emphasizing how polymer architectures influence surface reactivity, colloidal stability, and application efficiency in catalysis, environmental remediation, and biomedical contexts. The table serves as a concise reference for selecting and tailoring polymer-coated metal oxide nanocomposites for targeted applications.

## 5. Future Perspectives and Outlook

Polymer-coated metal and metal oxide nanoparticles are moving from isolated, case-by-case demonstrations toward deliberately designed, application-tailored materials. Advancing the field will require establishing quantitative links between polymer structure, grafting density, and shell permeability, and how these parameters collectively govern colloidal stability, reactivity, and biological behavior. More systematic comparisons between coating strategies, including “grafting to”, “grafting from”, “grafting through”, in situ, and layer-by-layer are needed to clarify how each approach affects polymer packing, interfacial robustness, and long-term function. To improve reproducibility and enable meaningful cross-study comparisons, the community would benefit from standardized benchmarks for coating thickness, grafting density, zeta potential, ion release profiles, and stability in relevant physiological or environmental conditions. From a manufacturing perspective, scalable and sustainable implementation will likely leverage aqueous or low-VOC (volatile organic compound) polymerizations, continuous-flow and photo-initiated “grafting from” processes, and click-based conjugations with integrated real-time quality control. Future coating designs should also adhere to safe-by-design principles, balancing stability against controlled degradation and clearance. Successful translation will require thorough evaluation of pharmacokinetics, biodistribution, cytocompatibility, sterilization compatibility, and shelf-life, to align with regulatory expectations. Looking forward, major opportunities include multifunctional theranostic platforms, flexible and antifouling biointerfaces, selective environmental remediation systems, and polymer-engineered energy nanomaterials. Integrating high-throughput synthesis, automated characterization, and data-driven optimization will accelerate the transition toward polymer-coated nanostructures with predictable, tunable, and scalable performance across biomedical, environmental, electronic, and energy applications.

## 6. Conclusions

This review provides a comprehensive analysis of polymer-coated metal and metal oxide nanoparticles, emphasizing their diverse structures and wide-ranging applications. The discussion categorizes these nanoparticles based on their inorganic core materials, including gold, silver, copper, platinum, palladium, iron oxide, titanium oxide, zinc oxide, and aluminum oxide. Each of these materials exhibits distinct properties, which are further enhanced by polymer coatings.

The multiple applications of these nanoparticles have been extensively explored, highlighting their roles in biosensing, photothermal therapy, targeted drug delivery, energy storage, environmental remediation, MRI, electronics, and antimicrobial treatments. In biomedical applications, these nanoparticles contribute to enhanced drug-delivery systems, improved imaging techniques, effective photothermal therapy, and higher MRI contrast resolution for more precise diagnostics. Their versatility is also evident in environmental applications, catalysts for pollutant degradation, and agents for water purification. Their antimicrobial properties make them particularly valuable for use in coatings and medical devices designed to prevent infections.

Beyond biomedical and environmental applications, polymer-coated metal and metal oxide nanoparticles are also critical in energy storage technologies, where they contribute to the development of supercapacitors, advanced batteries, and other energy storage devices. In electronics, they enhance the functionality of flexible circuits, conductive inks, and electronic components.

Additionally, this review underscores the critical role of polymer coatings in stabilizing metal nanoparticles, preventing aggregation, and protecting metal cores from oxidation, thereby extending their longevity in various industrial, environmental, biomedical, and energy-related applications. The review highlights how crucial it is to carefully select polymer materials, inorganic nanoparticles, and synthesis techniques to optimize the performance of these hybrid materials. The interaction between the polymer and inorganic nanoparticles is an important factor influencing stability, functionality, and overall performance of the nanocomposites. The choice of appropriate polymer coatings significantly impacts biocompatibility, surface reactivity, and physicochemical properties, which are essential for their effectiveness in specific applications.

In conclusion, this review demonstrates the versatility and broad applicability of polymer-coated metal and metal oxide nanoparticles, detailing their synthesis, characterization, and functionality across multiple scientific and technological domains. By emphasizing the unique properties of different nanoparticle types and their customized applications, this review highlights their critical role in advancing biomedical, environmental, and electronic technologies. Continued research and development in these hybrid materials will drive further innovations, making them indispensable in the advancement of next-generation nanotechnology and sustainable solutions.

## Figures and Tables

**Table 1:** Summary of polymer-coated metal nanoparticles, including metal cores, polymer coatings, synthesis methods, formation approaches as hybrid nanomaterials, and their applications.

Metal Core	Polymer Coating	Polymer Synthesis Method	Hybrid Nanomaterial Formation Mechanism	Applications	Ref.
AuNPs	cRGD-PEG-PLL-LA	Anionic	Grafting to	Drug delivery	[102]
AuNPs	lipoyl-[(MCH)_26_-*b*-(GMA)_53_]	RAFT	Grafting to	Drug delivery	[103]
AuNShs	P(NIPAM-*co*-AA)	Free radical	Grafting from	Drug delivery	[104]
AuNPs	poly(AcMan-*r*-AAm)	RAFT	Grafting to	Biosensing	[105]
AuNPs	Star-shaped PEG	N/A	Grafting to	Biosensing	[106]
AuNPs	DOPA, PEI	Self-polymerization, N/A	In situ	Environmental	[107]
AuNIs	PAH	N/A	Physisorption	Catalysis	[108]
AuNRs	PTEBS	N/A	Grafting to	Electronics	[109]
AuNPs	PEDOT:PSS, PVA	N/A	Grafting to	Electronics	[110]
AgNPs	PDA	Self-polymerization	Coordination	Antimicrobials	[111]
AgNPs	P(PEGMA-*co*-R-HEMA)	RAFT	Physisorption	Therapeutics	[112]
AgNPs	P(HEO_2_MA-*co*-MACPT)	RAFT	Grafting to	Drug delivery	[113]
AgNPs	PLL-PEG4-SPDP	N/A	Grafting to	Biosensing	[114]
AgNPs	PVP	N/A	In situ	Environmental	[115]
AgNPs	NFC-PVP	N/A	In situ	Electronics	[116]
CuNPs	PTP, PTPO	Free radical	In situ	Drug delivery	[117]
CuNPs	PANI	In situ	In situ	Antimicrobials	[118]
CuNPs	P4VP	Reversible ATRP	In situ	Environmental	[119]
PtNPs	Chitosan	N/A	In situ	Biosensing	[120]
PtNPs	PDDA, PSS	N/A	Layer-by-layer	Energy conversion	[121]
PdNPs	COS-RGD	N/A	Grafting to	Drug delivery	[122]
PdNPs	PAMAM dendrimer	Divergent method	In situ	Sensors	[123]

N/A denotes not applicable, as the information was not provided in the literature. Abbreviations: AuNPs—Gold nanoparticles, AuNShs—Gold nanoshells, AuNIs—Gold nanoislands, AuNRs—Gold nanorods, AgNPs—Silver nanoparticles, CuNPs—Copper nanoparticles, PtNPs—Platinum nanoparticles, PdNPs—Palladium nanoparticles, cRGD-PEG-PLL-LA—cyclic Arg-Gly-Asp (cRGD) peptide- poly(ethylene glycol)-*block*-poly(L-lysine)-lipoic acid, lipoyl-[(MCH)_26_-*b*-(GMA)_53_]—lipoyl-poly[2-(methacryloyloxy)ethyl-3-chloro-4-hydroxybenzoate]-*b*-[glycerol methacrylate], p(NIPAM-*co*-AA)—poly(*N*-isopropylacrylamide-*co*-acrylic acid), poly(AcMan-*r*-AAm)—poly(acrylamidophenyl α-mannose-*co*-acrylamide), PEG—poly(ethylene glycol), DOPA—dopamine, PDA—polydopamine, PEI—polyethyleneimine, PTEBS—poly[2-(3-thienyl)-ethyloxy-4-butylsulfonate], PEDOT:PSS—poly(3,4-ethylenedioxythiophene) polystyrenesulfonate, PVA—poly(vinyl alcohol), p(PEGMA-*co*-R-HEMA)—poly (ethylene glycol) methyl ether methacrylate-co-amino acid-methacrylate oxyethyl ester, p(HEO_2_MA-*co*-MACPT)—poly(2-(2-hydroxyethoxy)ethyl methacrylate-*co*-methacryloyloxy-3-thiahexanoyl-camptothecin), PLL-PEG4-SPDP—poly-L-lysine- poly(ethylene glycol)-succinimidyl 3-(2-pyridyldithio)propionate, PVP—poly(vinylpyrrolidone), NFC-PVP—nanofibrillated cellulose- poly(vinylpyrrolidone), PTP—poly(thymine-phosphate)methacrylate, PTPO—poly(thymine-phosphate-oligo(ethylene glycol)), PANI—polyaniline, P4VP—poly(4-vinylpyridine), PDDA—poly(diallyldimethylammonium chloride), COS-RGD—chitosan oligosaccharide-RGD peptide, PAMAM—poly(amidoamine), ATRP—atom transfer radical polymerization.

**Table 2:** Design guidelines for polymer-coated metal nanoparticles: representative core materials, polymer coatings, coating mechanisms, and key performance characteristics across major application domains.

Applications	Core Material	Polymer Coating	Coating Mechanism	Typical Size/Shell Thickness/Grafting Density	Stability/Cytotoxicity	Key Performance Notes	Ref.
Drug Delivery	AuNPs	cRGD-PEG-PLL-LA	Grafting to	Core: ~20 nm; polymer shell: ~8 nm	Stable in serum; no observed cytotoxicity	Targeted siRNA delivery; strong tumor inhibition	[102]
	AuNPs	lipoyl-[(MCH)_26_-*b*-(GMA)_53_]	Grafting to	Core: ~15–20 nm; polymer shell ~10–12 nm at pH 7.4, shrinking to ~5–7 nm at pH 6.5	Stable in physiological media; no significant cytotoxicity in KB and MCF-7 cells	pH-triggered “hide-and-reveal” folate targeting; significantly higher uptake at pH 6.5 compared to pH 7.4	[103]
	AuNShs	P(NIPAM-*co*-AA)	Grafting from	Core: ~120 nm; polymer shell: ~20–30 nm	Reversible collapse at LCST (~40 °C)	NIR-responsive heating and on-demand drug release	[104]
	AgNPs	P(HEO_2_MA-*co*-MACPT)	Grafting to	Core: ~50 nm; polymer shell: ~10 nm	Stable at pH 7.4; release triggered in acidic environments	Monitored CPT delivery via fluorescence “off/on” signal	[113]
	CuNPs	PTP, PTPO	In situ	PTP- and PTPO-stabilized NPs: ~20–30 nm in TEM	pH/GSH-responsive release with reduced side effects	Dual chemo + chemodynamic therapy in GBM	[117]
	PdNPs	COS-RGD	Grafting to	Core: ~22 nm; polymer shell: ~2–3 nm	Stable under physiological conditions; pH-triggered drug release; low systemic toxicity in mouse models	RGD-mediated tumor targeting and strong NIR photothermal response; significant tumor suppression in vivo	[122]
Biosensing	AuNPs	poly(AcMan-*r*-AAm)	Grafting to	Core: ~40 nm; polymer shell: ~6–14 nm	Salt-responsive colloidal stability	Multivalent mannose-driven ConA recognition	[105]
	AuNPs	Star shaped PEG	Grafting to	Core: ~19 nm; polymer shell: ~25–30 nm	Stable in buffer due to PEG steric repulsion	4-arm PEG yields stronger IL-8 sensing response than 8-arm PEG	[106]
	AgNPs	PLL-PEG4-SPDP	Grafting to	Core: ~80 nm; polymer shell: ~4 nm in TEM	Stable dispersion	26x fluorescence enhancement; high sensitivity biosensing	[114]
	PtNPs	Chitosan	In situ	Ch-PtNPs: ~2 nm	Biocompatible due to chitosan coating	ACP sensing, LOD ~0.016 U·L^−1^	[120]
Environmental	AuNPs	DOPA, PEI	In situ	N/A (No size information)	Stable under continuous use, low NP leaching	High catalytic efficiency for PNP and dye degradation	[107]
	AgNPs	PVP	In situ	PVP-coated AgNPs: ~40–50 nm increasing PVP % reduces size)	Stable and well-dispersed; effective against *E. coli* and *S. aureus*	Water purification; effective bacteria removal	[115]
	CuNPs	P4VP	In situ	Core CuNPs: ~10 nm; HNTs: 0.5–2 μm length; inner diameter 20–30 nm; shell thickness 15–20 nm	Minimal leaching; stable antibacterial action	94.5% bacteriostasis vs. *E. coli*, enhanced membrane hydrophilicity and water flux; suitable for water purification	[119]
Electronics	AuNRs	PTEBS	Grafting to	Core NRs: ~25 nm (short axis) × ~110 nm (long axis); ligand shell thickness: ~0.7–2.1 nm	Stable in polar media; 1+ year conductivity retention	Sinter-free inks; resistivity ~10^−6^–10^−7^ Ω·m; flexible printed films	[109]
	AuNPs	PEDOT:PSS, PVA	Grafting to	Core NPs: 47 nm; film thickness with PVA: 0.4 to 1.0 μm	Stable 280 days; low resistivity; strong adhesion	High conductivity (2.1 × 10^5^ S·m^−1^); sinter-free; flexible	[110]
	AgNPs	NFC-PVP	In situ	PVP-AgNPs: ~25 nm	Stable dispersion within polymer network; no aggregation reported	Improved conductivity and flexibility for wearable electronics	[116]
Antimicrobials	AgNPs	PDA	Coordination	Core PVP-AgNPs: ~32 nm; PDA-capped AgNPs: ~36–54 nm	Stable; ROS-enhanced antibacterial action	Enhanced antibacterial action via PDA-mediated ROS	[108]
	CuNPs	PANI	In situ	CuNPs ~6 nm uniformly dispersed in PANI matrix	PANI prevents CuNP aggregation; stable dispersion	Strong antimicrobial activity; synergistic membrane-disruptive effects	[118]
Therapeutics	AgNPs	P(PEGMA-*co*-R-HEMA)	Physisorption	Ag core: ~16 nm; polymer-coated sizes: ~28–51 nm depending on amino acid	Stable colloidal dispersion	Inhibits and reverses insulin fibrils	[112]
Catalysis	AuNIs	PAH	Physisorption	PAH layer thickness: 0.54 nm	Stable coating; maintains AuNI morphology	Enhanced catalytic activity via electrostatic enrichment of anionic reactants	[108]
Energy Conversion	PtNPs	PDDA, PSS	Layer-by-layer	Pt core: ~2.6 nm; PDDA-capped NPs: ~11 nm	Uniform film formation in LbL assemblies	Efficient HER electrocatalysis	[121]
Sensors	PdNPs	PAMAM dendrimer	In situ	N/A (No size information)	Stable immobilization via dendrimer anchoring; magnetic recyclability	H_2_O_2_ sensing; linear range 0.05–160 μM; LOD 0.01 μM	[123]

Abbreviations are defined in Table 1 and upon their first appearance in the text.

**Table 3:** Summary of polymer-coated metal oxide nanoparticles, including metal oxide cores, polymer coatings, synthesis methods, formation approaches as hybrid nanomaterials, and their applications.

Metal Oxide Core	Polymer Coating	Polymer Synthesis Method	Hybrid Nanomaterial Formation Mechanism	Applications	Ref.
Fe_3_O_4_ NPs	PAC	Free radical	Grafting through	Drug delivery	[165]
Fe_3_O_4_ NPs	CN	N/A	Layer-by-layer	Drug delivery	[166]
Fe_3_O_4_ NPs	PEG/PEI/Tween 80	N/A	In situ	MRI	[167]
Fe_3_O_4_ NPs	PS-*b*-PDMS	ATRP	Grafting to	MRI	[168]
Fe_3_O_4_ NPs	PS-*b*-PAA, PS-PEO-SH	N/A, RAFT	Grafting to	MRI-guided drug delivery	[169]
Fe_3_O_4_ NPs	CFP, R-PEI	ROP, N/A	Grafting to	Gene delivery	[170]
Fe_3_O_4_ NPs	PNIPAAM	RAFT	Grafting from	Hyperthermia	[171]
TiO_2_ NPs	PANI	Oxidative chemical polymerization	Physisorption	Bone tissue regeneration	[172]
TiO_2_ NPs	PTH	Oxidative chemical polymerization	Grafting from	Biomedical devices	[173]
ZnO NPs	Chitosan	N/A	Physisorption	Antibacterials	[174]
ZnO NPs	Borate- and methacrylate-based polymer films	Plasma polymerization	N/A	Automotive industry	[175]
Al_2_O_3_ NPs	γ-PGA	N/A	Physisorption	Cancer treatment	[176]
Al_2_O_3_ NPs	PTFE	N/A	N/A	Antibacterials	[177]
Al_2_O_3_ NPs	Chitosan	N/A	Physisorption	Catalysis	[178]

N/A denotes not applicable, as the information was not provided in the literature. Abbreviations: Fe_3_O_4_ NPs—iron oxide nanoparticles, TiO_2_ NPs—titanium dioxide nanoparticles, ZnO NPs—zinc oxide nanoparticles, Al_2_O_3_ NPs—aluminum oxide nanoparticles, PAC—poly(*N*-isopropylacrylamide-acrylamide-chitosan), CN—casein, PEG/PEI/Tween 80—polyethylene glycol/polyethylene imine/polysorbate 80, PS-*b*-PDMS—polystyrene-*b*-poly(dimethylsiloxane), PS-*b*-PAA—poly(styrene)-*b*-poly(acrylic acid), PS-PEO-SH—thiol-ended poly(styrene)-*b*-poly(ethylene oxide), CFP—catechol-functionalized polypeptide, R-PEI—rhodamine-labeled polyethyleneimine, PNIPAAM—poly(*N*-isopropylacrylamide), PANI—polyaniline, PTH—polythiophene, γ-PGA—poly(γ-glutamic acid), PTFE—polytetrafluoroethylene, ATRP—atom transfer radical polymerization, RAFT—reversible addition-fragmentation chain transfer, ROP—ring-opening polymerization, MRI—magnetic resonance imaging.

**Table 4:** Design guidelines for polymer-coated metal oxide nanoparticles: representative core materials, polymer coatings, coating mechanisms, and key performance characteristics across major application domains.

Applications	Core Material	Polymer Coating	Coating Mechanism	Typical Size/Shell Thickness/Grafting Density	Stability/Cytotoxicity	Key Performance Notes	Ref.
Drug delivery	Fe_3_O_4_ NPs	PAC	Grafting through	Core: ~10 nm; polymer shell: ~70 nm (~150 nm final)	No significant cytotoxicity up to 500 μg·mL^−1^	Dual-responsive DOX release (heat and pH); R11 peptide enhances PC3 cell uptake	[165]
	Fe_3_O_4_ NPs	CN	Layer-by-layer	Core: ~10 nm; hydrodynamic diameter ~24 nm (shell: ~7–9 nm)	Stable in gastric pH; enzyme-responsive	Enhanced oral DOX absorption	[166]
MRI	Fe_3_O_4_ NPs	PEG/PEI/Tween 80	In situ	PEG-SPIONs: ~9 nm; PEG/PEI/Tween 80-SPIONs: ~11–12 nm	Stable in water; PEG slows clearance and reduces toxicity	PEG-SPIONs provide enhanced MRI brain contrast and prolonged circulation	[167]
	Fe_3_O_4_ NPs	PS-*b*-PDMS	Grafting to	Core: ~15 nm; polymer shell increases interparticle spacing to ~21 nm	pH-responsive morphology	Janus pupa-like particles provide enhanced *T*_2_ MRI contrast	[168]
Antibacterials	ZnO NPs	Chitosan	Physisorption	Rod-like grains ~20–150 nm (FE-SEM)	Stable in aqueous media; strong antibacterial effect	Photocatalytically degrades congo red and methylene blue under sunlight	[174]
	Al_2_O_3_ NPs	PTFE	N/A	Al_2_O_3_ NPs: ~50 nm	Stable dispersion (zeta potential: ~+50 mV); low cytotoxicity to fibroblasts	Generates ROS and inhibits *E. coli* growth; suitable for food-contact antibacterial coatings	[177]
MRI-guided drug delivery	Fe_3_O_4_ NPs	PS-*b*-PAA, PS-PEO-SH	Grafting to	Membrane thickness tunable ~10–93 nm (monolayer-multilayer vesicles)	Stable colloidal dispersions, superparamagnetic behavior retained	Controlled release via membrane thickness; RGD functionalization enabled tumor targeting and MRI-guided therapy	[169]
Gene delivery	Fe_3_O_4_ NPs	CFP, R-PEI	Grafting to	Oleic acid-capped Fe_3_O_4_ NPs: ~15 nm; with polymer shell: ~40 nm	Stable, positively charged in aqueous media	Efficient gene delivery; MRI and fluorescence cell tracking	[170]
Hyperthermia	Fe_3_O_4_ NPs	PNIPAAM	Grafting from	RAFT agent-capped NPs: ~19 nm; polymer layer: ~1.5 nm thick	LCST tunable above 37 °C (stable at body temperature; collapses under hyperthermia)	Heat-triggered or pH-triggered DOX release; retains superparamagnetism; >90% cell viability	[171]
Bone tissue regeneration	TiO_2_ NPs	PANI	Physisorption	Fibers ~0.5–0.8 μm (diameter varies with TiO_2_/PANI loading)	Stable, bioactive surface	Enhances osteogenesis and controlled SIM release	[172]
Biomedical devices	TiO_2_ NPs	PTH	Grafting from	PTH-TiO_2_ nanocomposite: ~70–90 nm	Maintains ~88% activity (14 days)	Enhanced GOx-mediated bioanode performance; improved electron transport in BFCs	[173]
Automotive industry	ZnO NPs	Borate- and methacrylate-based polymer films	N/A	ZnO NPs: ~10–30 nm	Stable under high load/heat	~95% wear reduction	[175]
Cancer treatment	Al_2_O_3_ NPs	γ-PGA	Physisorption	Core: ~5 nm, polymer-coated: ~7 nm	Stable, negatively charged dispersion; enhanced cancer cell cytotoxicity (PC-3)	Selective adsorption of positively charged proteins (e.g., lysozyme); ROS-mediated cancer cell killing	[176]
Catalysis	Al_2_O_3_ NPs	Chitosan	Physisorption	Al_2_O_3_ NPs: ~50 nm	Reusable catalyst up to ≥4 cycles with minimal activity loss	Enhanced base-catalyzed synthesis yield; heterogeneous, green catalyst enabling easy separation	[178]

Abbreviations are defined in Table 3 and upon their first appearance in the text.

## Data Availability

No new data were created or analyzed in this study.
